# *ZCF32*, a fungus specific Zn(II)2 Cys6 transcription factor, is a repressor of the biofilm development in the human pathogen *Candida albicans*

**DOI:** 10.1038/srep31124

**Published:** 2016-08-08

**Authors:** Pallavi Kakade, Parag Sadhale, Kaustuv Sanyal, Valakunja Nagaraja

**Affiliations:** 1 Department of Microbiology and Cell Biology, Indian Institute of Science, C.V. Raman Avenue, Bangalore 560012, India; 2 Jawaharlal Nehru Centre for Advanced Scientific Research, Bangalore 560064, India

## Abstract

As a human fungal pathogen, *Candida albicans* can cause a wide variety of disease conditions ranging from superficial to systemic infections. Many of these infections are caused by an inherent ability of the pathogen to form biofilms on medical devices resulting in high mortality. Biofilms formed by *C. albicans* are a complex consortium of yeast and hyphal cells embedded in an extracellular matrix and are regulated by a network of transcription factors. Here, we report the role of a novel Zn(II)2-Cys6 binuclear cluster transcription factor, *ZCF32,* in the regulation of biofilm formation. Global transcriptome analysis reveals that biofilm development is the most altered pathway in the *zcf32* null mutant. To delineate the functional correlation between *ZCF32* and biofilm development, we determined the set of genes directly regulated by Zcf32. Our data suggests that Zcf32 regulates biofilm formation by repressing the expression of adhesins, chitinases and a significant number of other GPI-anchored proteins. We establish that there is the lesser recruitment of Zcf32 on the promoters of biofilm genes in biofilm condition compared to the planktonic mode of growth. Taking together, we propose that the transcription factor *ZCF32* negatively regulates biofilm development in *C. albicans*.

*Candida albicans* (*C. albicans*) is the fourth most leading cause of hospital-acquired infections and probably the most frequently isolated fungus from patients^1^,^2^. It is a normal flora organism of humans residing in the epithelial lining of the gut, respiratory tract, and female genito-urinary tract. In spite of being a commensal, it is capable of causing varied levels of disease conditions ranging from superficial to systemic infections in immunocompromised hosts, proving itself as a successful opportunistic pathogen^2^. The infections are often caused by an inherent ability of the pathogen to form a robust biofilm on a wide variety of medical devices such as venous catheters, urinary catheters, pacemakers and artificial joints with over 10 million recipients per year. *C. albicans* infections associated with ICU patients result in 30–50% mortality^3^. Biofilms are the surface associated structures consisting of a population of microorganisms embedded in extracellular polymeric substances^4^. Biofilm formed by *C. albicans* is a complex consortium of yeast, hyphae and extracellular matrix produced by these cells. *C. albicans* can also form biofilms *in vivo* on tissue surfaces of oral and vaginal mucosa^5^.

A number of genes implicated in regulating biofilm formation are putative transcription factors modulating the biofilm formation directly or indirectly. Six master regulators - *BCR1, BRG1, EFG1, NDT80, ROB1* and *TEC1* regulate the biofilm development in *C. albicans*^6^. Each master regulator also controls the expression of another master regulator, resulting in a complex and intertwined regulatory circuit. Together, the six master regulators directly bind to approximately 1,000 target genes, many of which belong to transcription factor families^6^. In addition, 44 transcription factors have been identified which are important for biofilm development and are directly regulated by one of the six master regulators^7^. More recently, Fox *et al.* have identified additional major regulators of biofilm formation which include *FLO8, GAL4, RFX2*^8^. Most of these transcription factors positively regulate the biofilm formation. To date, only a few transcription regulators, *UME6*, *NRG1*, *GAL4, RFX2, SFP1,* and *ZAP1* have been reported to have a negative impact on the biofilm pathway^8^,^9^,^10^,^11^. In addition to the transcription factors, a number of genes belonging to various functional categories have been shown to participate in biofilm development. *YWP1*, *ADH1*, *IFD6*, *CHK1,* and *TOR1*^6^,^12^,^13^,^14^ are shown to have a negative impact on biofilm formation while a large number of genes exert a positive effect^7^.

*In vitro* biofilm development is considered to be a stepwise process. Adhesion is the first and crucial step during biofilm growth and the role of various cell wall proteins and transcription factors in the process has been demonstrated^15^,^16^,^17^,^18^,^19^. Over 10% of *C. albicans* transcription factors are reported to govern adhesion^15^. Further, 37 cell surface proteins are under the regulation of 12 transcription factors forming a regulon which govern adhesion in response to environmental cues^15^. Amongst the six master regulators, *BCR1* and a subset of its downstream target genes, *ALS1*, *ALS3* and *HWP1* are required for adherence^4^,^17^,^20^. Many different kinds of adhesins are shown to play vital roles in the process of adhesion and thus biofilm formation. Mutant strains defective in adhesion do not form a proper biofilm^15^. Adhesins contribute to cell-surface as well as cell-cell adhesion. The second step in biofilm formation is the yeast to hyphae transition. Hyphae are important for adherence to yeast cells and other hyphal cells by which they provide structural integrity and stability to the biofilm^20^. Mutants lacking the ability to transit from one form to another are reported to form defective biofilms. Many studies have shed light on the various genes regulating yeast to hyphae transition and biofilm formation in *C. albicans*^11^,^21^,^22^. The master regulators *EFG1*, *TEC1*, *NDT80*, *ROB1* and a few of their downstream target genes are required for proper hyphal development^6^. The third step in biofilm development is the production of the extracellular matrix (ECM) which is followed by dispersion of yeast cells. Unlike the previous steps, little is known about the regulation and production of ECM as well as regulation of the dispersion of yeast cells. The only reported transcription regulators of ECM production are *ZAP1* which controls the process negatively and *RLM1* which regulates it in a positive fashion. Two transcriptional regulators of biofilm dispersal have been identified, Nrg1 and Ume6, which negatively govern the process^10^,^11^.

Here, we report the role of a novel transcription factor, *ZCF32*, in the repression of biofilm development. Orf19.5940 has been annotated as *ZCF32* as it possesses Zn(II)2 Cys6 DNA binding domain. The genome-wide expression analysis, the selective evolution of ligands by exponential enrichment (SELEX) and ChIP- qPCR data revealed that *ZCF32* negatively regulates biofilm formation. We show that this newly identified transcription factor negatively regulates the adhesion and filamentation processes by repressing the expression of adhesins and various cell wall proteins. Further, more recruitment of Zcf32 on the promoters of biofilm genes is observed in the planktonic condition as compared to the biofilm condition confirming the negative impact of *ZCF32* on the biofilm pathway.

## Results

### Identification of *ZCF32* regulated genes

*ZCF32* was found to be upregulated in the microarray carried out with the oral thrush samples collected from patients suffering from oral candidiasis (unpublished data). It belongs to Zn(II)2 Cys6 binuclear cluster transcription factor family^23^,^24^. The presence of Zn(II)2 Cys6 binuclear cluster transcription factors has been shown to be confined to the fungal kingdom only^25^,^26^,^27^. The homologs of these proteins are absent in prokaryotes and higher eukaryotes. The mRNA level of expression of *ZCF32* in cells grown till early log phase was found to be very low. However, its expression increased in a time-dependent manner, reaching maxima at the start of the stationary phase and then decreasing subsequently (Supplementary Fig. S1A,B). To elucidate the role of *ZCF32,* it was deleted from *C. albicans* wild-type SC5314 strain using the SAT1 flipper cassette strategy^28^ and the desired homozygous mutants were identified by Southern blot analysis (Supplementary Fig. S1C). The homozygous null mutant of *zcf32* was viable suggesting that *ZCF32* is not essential for the viability of *C. albicans.* Growth rates of wild-type and homozygous mutant were compared by optical density measurement (Supplementary Fig. S2A) and CFU analysis (Supplementary Fig. S2B) at different intervals of time in YPD medium at 30 °C. The growth rates were observed to be similar. Thus, *ZCF32* does not affect the growth of *C. albicans* in laboratory conditions.

**Figure 1:**
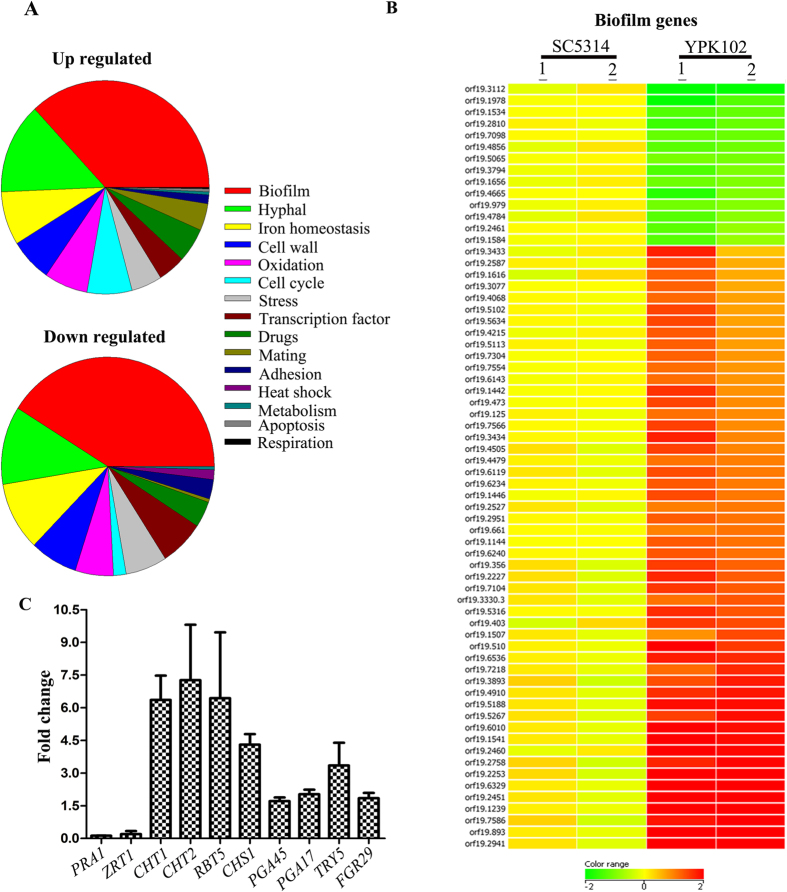
Transcriptome analysis revealed the possible role of *ZCF32* in biofilm development. (**A**) Transcriptome profiles of SC5314 (wild-type) and YPK102 (*zcf32*/*zcf32*) were compared by microarray analysis. Both the strains were grown in YPD at 30 °C till the start of the stationary phase. A total of 607 genes were found to be differentially regulated in the mutant as compared to the wild-type. Pie charts depict the functional categorization of upregulated (438 genes) and downregulated (169 genes) genes in YPK102. The maximum numbers of genes with altered expression from both the groups belong to the biofilm pathway followed by yeast to hyphal transition pathway (p-value ≤ 0.05). (**B)** Expression data (≥2 fold difference with p-value ≤ 0.05) of biofilm-related genes from wild-type and mutant is illustrated as the heat map. Gene expression values are represented in the form of a colour coded scale as shown at the bottom of the heat map. (**C)** Validation of differentially expressed genes in *zcf32* null mutant (YPK102) was carried out by quantitative PCR analysis. The SC5314 and YPK102 strains were grown independently three times in YPD at 30 °C till the start of the stationary phase. RNA was extracted from each strain followed by cDNA synthesis and expression analysis of a few cell wall proteins and adhesins was carried out by real-time PCR. All the samples were normalized to *ACT1* gene control.

To delineate the role of *ZCF32* in *C. albicans* biology, we carried out microarray with SC5314 versus YPK102 (*zcf32*/*zcf32*) strains grown at 30 °C. Since *ZCF32* showed maximum expression at the start of the stationary phase, the cells were harvested at the stationary phase of growth. In the microarray analysis, a total of 607 genes were found to be expressed significantly different (≥1.5 fold change in expression level and p-value ≤ 0.05) in the YPK102 as compared to the wild-type (Supplementary Fig. S3). Out of 607 genes with altered expression, 438 genes were found to be upregulated in the mutant while 169 showed downregulation (Supplementary sheet 1). Gene ontology analysis (p-value ≤ 0.05) of the altered genes showed a maximum number of differentially expressed genes to be biofilm-related followed by the genes involved in hyphae formation (Fig. 1A, Supplementary sheet 2).

Out of 607 differentially expressed genes in YPK102, 253 were found to be involved in the development of biofilm. Among 253 biofilm-related genes, 167 genes were upregulated while 86 were downregulated in YPK102. A few of the biofilm genes showing significant upregulation or downregulation are shown in Fig. 1B. Transcriptome analysis showed that an adhesin*- TRY5* and many cell wall proteins-*RBT5, CHT1, CHT2, CHT3, CHS1, PGA45, PGA17* and *FGR29* were upregulated in YPK102, implying that *ZCF32* is a negative regulator of these genes while zinc homeostasis genes, *PRA1* and *ZRT1* were highly repressed in the mutant suggesting that they are under positive regulation of *ZCF32* (Table 1). Differential regulation of these genes in the YPK102 was further validated by qRT-PCR analysis (Fig. 1C). These sets of genes (Table 1) have been earlier reported to be altered in the biofilm mode of growth^29^,^30^.

### *ZCF32* mutants form enhanced biofilm

**Figure 2:**
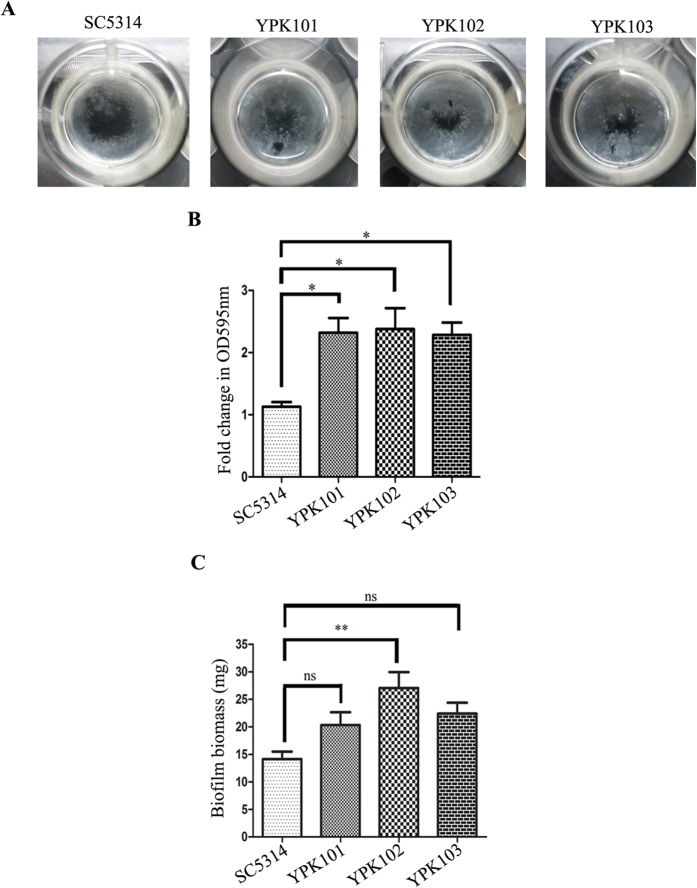
The absence of *ZCF32* results in an enhanced biofilm formation. (**A**) The visual appearance of biofilms developed in 12 well plate using Spider medium till 60 h. Biofilm formed by YPK102 appeared denser than SC5314 biofilm. (**B)** SC5314, YPK101, YPK102 and YPK103 strains were grown in biofilm mode in 12-well plates for 60 h and quantitation of total biofilm was carried out by the crystal violet staining assay. The experiment was carried out three times independently and results are plotted as the standard error of mean (SEM) using Graph Pad Prism5 (*p-value ≤ 0.02). Statistical significance was calculated using one-way analysis of variance (ANOVA) coupled with Bonferroni’s test. (**C)** Biofilm biomass determination was done by the dry mass measurement for SC5314, YPK101, YPK102, and YPK103. Strains were grown in standard biofilm conditions for 60 h. Results from three sets of independent experiments were considered and statistical significance was calculated using one-way ANOVA coupled with Bonferroni’s post test. Results are shown as SEM (**p-value ≤ 0.02).

To understand the role of *ZCF32* in biofilm formation, *in vitro* assays of biofilm development were carried out for SC5314 (wild-type), *ZCF32*/*zcf32* (YPK101)*, zcf32/zcf32* (YPK102) and *zcf32*/*zcf32::ZCF32* (YPK103) strains (Supplementary Table S3). All the four strains were allowed to form biofilm in 6 well polystyrene plates in Spider medium and it was observed that YPK102 formed denser biofilm as compared to the wild-type (Fig. 2A). Quantitative differences in biofilm formation of wild-type and *zcf32* mutants were assessed by performing crystal violet staining and dry mass measurement assays of biofilms grown for 60 h. Crystal violet staining of mature biofilms indicated that YPK102 mutant exhibited more than 2-fold increase in the biofilm formation as compared to the wild-type (Fig. 2B) implying enhanced biofilm formation in the absence of *ZCF32*. Further, to gain an insight into the overall changes in biofilm formation, SC5314, YPK101, YPK102 and YPK103 were subjected to dry mass measurement. YPK102 mutant showed highest biofilm biomass, followed by intermediate biofilm biomass production by *ZCF32* heterozygous and single re-integrant strains. Wild-type strain showed the least amount of biofilm biomass production (Fig. 2C). These results manifest that the absence of *ZCF32* leads to an increased biofilm production, further revealing its repressive role in biofilm development.

### *ZCF32* negatively regulates biofilm development by repressing adhesion, yeast to hyphae transition and dispersion

**Figure 3:**
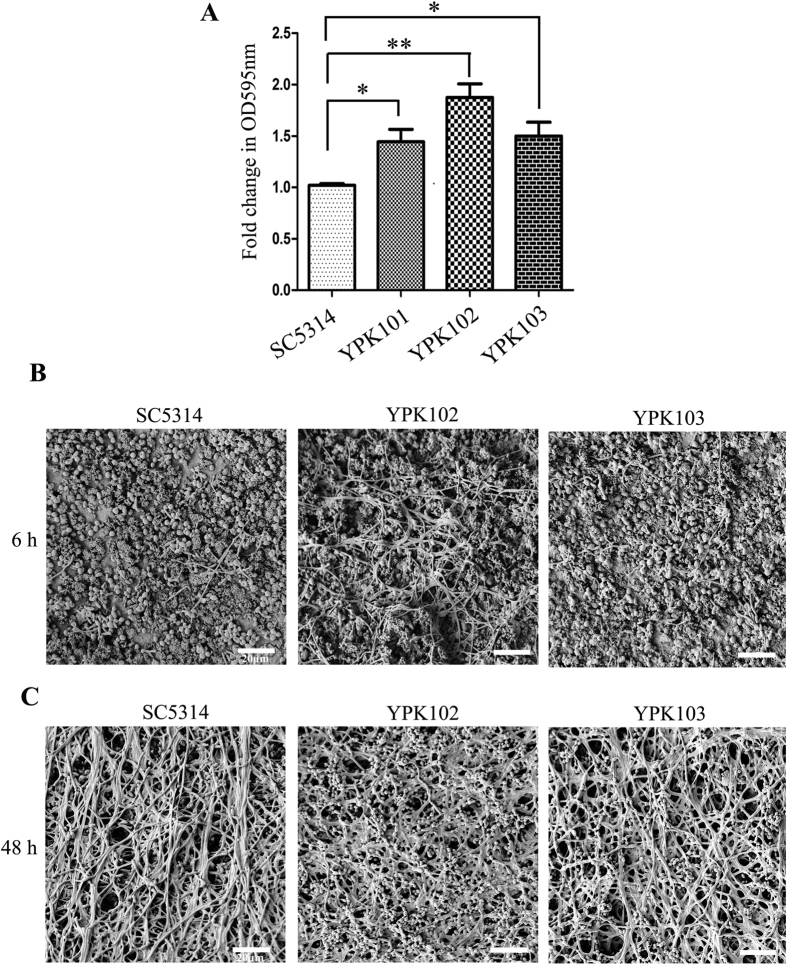
*ZCF32* negatively regulates biofilm formation by repressing adhesion, yeast to hyphae transition and dispersion. (**A**) The adhesion assay was carried out by crystal violet staining of the yeast cells adhered to the bottom of the polystyrene plate. Results from three independent experiments were considered for the statistical analysis. Statistical significance (p-value) was calculated with a Student’s one-tailed paired t-test and is represented by the asterisks (*p value ≤ 0.02, **p-value ≤ 0.002). (**B**) Biofilms were grown *in vitro* on human urinary catheter till 6 h (early biofilm) to score for hyphae formation. Catheter pieces with biofilms formed by SC5314, YPK102, and YPK103 strains were analyzed by scanning electron microscopy (SEM). Representative images of each strain are shown. Scale bar (white line) 20 μm. **C)** To study the dispersion ability of different strains, the presence of yeast cells on the surface of 48 h old biofilm (mature biofilm) was scored. Surface topographies of biofilms formed by SC5314, YPK102, and YPK103 strains were analyzed by SEM. Scale bar (white line) 20 μm.

To understand the steps in biofilm development that are regulated by *ZCF32*, we first assayed for the yeast cells adhered to the bottom of polystyrene plates. Different strains under study were subjected to adherence for 2 h followed by crystal violet staining of the adhered cells. The results show that the YPK102 mutant exhibits 1.8–2 fold more adherence as compared to the reference strain (Fig. 3A). The introduction of a copy of *ZCF32* in the null mutant rescued the phenotype partially, implying that deletion of *ZCF32* enhances adhesion to the polystyrene plate. Thus, *ZCF32* is a repressor of the adhesion process.

The second step of biofilm formation involves the transition of yeast cells to hyphal cells. We compared the extent of hyphae formation in the biofilm mode of growth by scanning electron microscopy (SEM) and confocal laser scanning microscopy (CLSM) analysis. Biofilms were grown for 6 h either on the human urinary catheter for SEM studies or on silicon sheets for CLSM studies. SEM and CLSM analyses of early biofilm exhibited that YPK102 null mutant forms more robust hyphae as compared to the wild-type and single copy complemented strains, indicating that the transcription factor indeed regulates yeast to hyphae transition in a negative manner (Fig. 3B and Supplementary Fig. S4).

The presence of yeast cells on the surface of the mature biofilm is a hallmark of dispersion^10^. Hence, we studied the dispersion ability of different strains by comparing the surface topography of mature biofilms. SEM imaging of 48 h old biofilms grown on human urinary catheter showed the presence of more yeast cells on biofilm surface of the null mutant compared to the wild-type strain (Fig. 3C). The re-integrant strain with one copy of *ZCF32* showed the intermediate phenotype. Dispersion of yeast cells helps in the establishment of biofilm structures at different niches in the host as well as on different parts of implanted medical devices thus completing the biofilm development cycle^29^,^31^. Since a higher number of yeast cells are present on the surface of *zcf32* null mutant compared to SC5314 in the mature stage of biofilm growth, it implies that these cells might lead to more dispersion and thus faster and more robust biofilm formation in the case of *zcf32* mutant compared to the wild-type. Considering all these results, we propose that *ZCF32* negatively regulates biofilm development by repressing adhesion, yeast to hyphae transition and dispersion.

### Zcf32 binds to DNA in a sequence-specific manner

**Figure 4:**
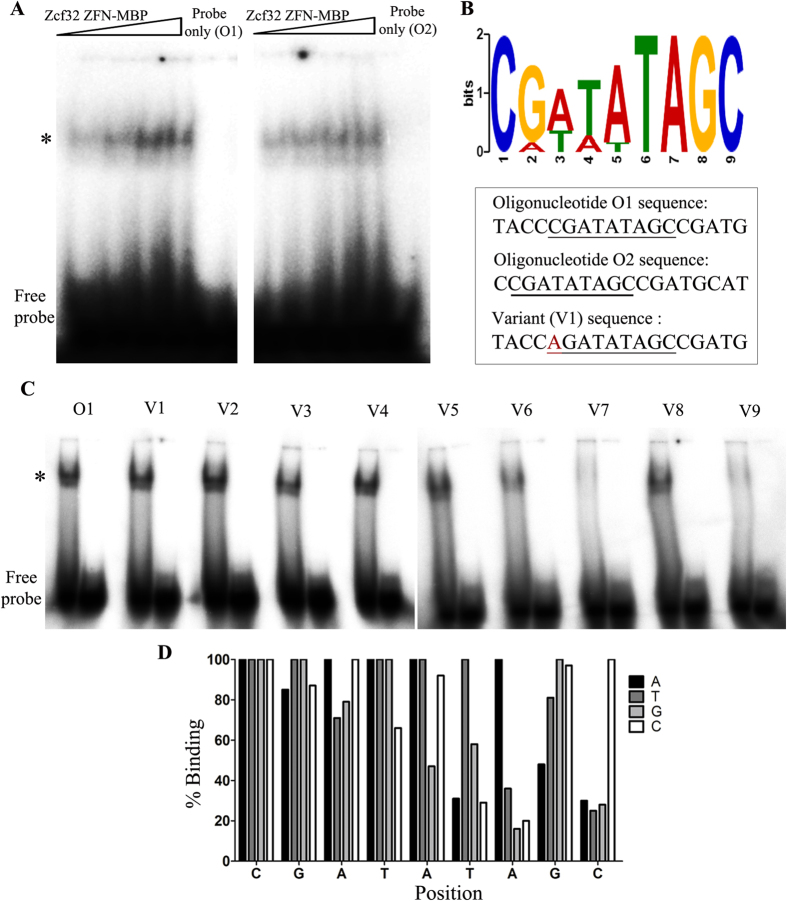
SELEX revealed the sequence-specific binding of Zcf32 to double-stranded DNA. (**A**) An electrophoretic mobility shift assay (EMSA) was performed using purified Zcf32 ZFN-MBP fusion protein (with increasing protein concentrations as indicated) and ATP-[γ-^32^P] labelled 18 bp double-stranded DNA oligonucleotides 1 and 2 (O1- TACCCGATATAGCCGATG and O2- CCGATATAGCCGATGCAT). The mobility shift is illustrated by an asterisk. (**B**) SELEX revealed that Zcf32 binds to eighteen independent double-stranded DNA sequences. A consensus analysis was carried out for these DNA sequences using MEME analysis tool and a 9 bp long consensus logo was obtained. Below the logo, sequences of the 18 bp long ds DNA oligonucleotides O1, O2 and the variant V1 are shown wherein the consensus region is underlined. In the case of the variant oligonucleotides, the mutated base is highlighted in red colour. (**C**) An EMSA was carried out with oligonucleotide O1and variant oligonucleotides (V1 to V27) to analyze the critical positions in the binding consensus of Zcf32. The binding ability of recombinant Zcf32 ZFN-MBP protein was analyzed for 27 variant oligonucleotides. Two representative EMSA gels for O1 and variant oligonucleotides V1 to V9 are shown. Asterisk indicates the shift in the mobility of ATP-[γ-^32^P] labelled ds DNA. The second lane for each oligonucleotide sequence in the EMSA gel indicates the probe (ATP-[γ-^32^P] labelled ds DNA) control. (**D**) Summary of the results from the mutational analysis of Zcf32 binding consensus sequence is represented in the form of a bar graph. The intensity of the shifted DNA was measured using Multigauge tool for each reaction carried out. The intensity of the shifted band obtained from variant DNA oligonucleotide was divided by the same of O1 oligonucleotide, thus giving the fraction of protein bound to the particular oligonucleotide sequence. Finally, the data is presented as percent binding of Zcf32 ZFN-MBP to O1and variant oligonucleotides in the form of a bar graph. Mutational analysis with single base pair change at a particular position shows that T, A and C at positions 6, 7 and 9 respectively are critical for the binding of Zcf32 ZFN domain.

*ZCF32* has been annotated as a putative Zn(II)2Cys6 DNA binding transcription factor^23^. To decipher the DNA binding property of this protein, SELEX experiment was carried out. An N-terminal 348 bp of *ZCF32* containing the Zn(II)2Cys6 binuclear cluster domain was cloned in pMAL-c2x (NEB). Zcf32 ZFN-MBP, as well as MBP (control), were expressed, purified and SELEX was performed with a library of 54 bp double-stranded DNA oligonucleotides till the seventh round. Saturation in the level of enriched double-stranded DNA (ds DNA) was observed after the sixth round of SELEX (Supplementary Fig. S5A). Binding of the fusion protein to the 54 bp ds DNA oligonucleotides enriched in the SELEX was confirmed by electrophoretic mobility shift assay (EMSA) (Supplementary Fig. S5B). Further, the seventh round enriched oligonucleotides were cloned into the pGEM-T Easy cloning vector. From the sequencing of 60 positive clones, eighteen independent DNA sequences were obtained with variable frequencies of occurrence (Table 2). The specific binding of Zcf32 ZFN-MBP to the core 18 bp sequence (excluding the M13 forward and M13 reverse primer sequences) within the 54 bp ds DNA sequence was further validated by EMSA (Fig. 4A). Binding of Zcf32 ZFN-MBP with two different oligonucleotide sequences O1- TACCCGATATAGCCGATG and O2- CCGATATAGCCGATGCAT was tested by EMSA. A 9 bp long consensus logo was obtained from eighteen independent DNA sequences by multiple em for motif elicitation (MEME) analysis tool (Fig. 4B).

To identify the critical base pairs required for Zcf32 binding, mutational analysis of the most frequently occurred oligonucleotide, O1 was carried out. The 9 bp consensus sequence is a part of O1. Each base position in the 9 bp sequence was changed to remaining three bases, one at a time. EMSA reactions were carried out for Zcf32 ZFN-MBP protein with total 27 variant ds DNA oligonucleotides (Fig. 4C and Supplementary Fig. S6A,B). Mutational analysis data showed that C, G and A at positions 1, 2 and 3 respectively, were not crucial for Zcf32 binding as the mutation of these bases did not hamper the binding of the protein (Fig. 4D). However, the mutations of T, A and C at positions 6, 7 and 9 respectively, reduced the protein binding significantly, ascertaining the critical nature of these base positions. A summary of the results of the mutational analysis is showed in Fig. 4D. Thus, these results unveil that Zcf32 specifically recognises the ds DNA sequence and 6 bp core sequence is crucial for its binding.

### *In silico* genome-wide analysis of Zcf32 binding sites exhibits preferential presence on the promoters of biofilm genes

**Figure 5:**
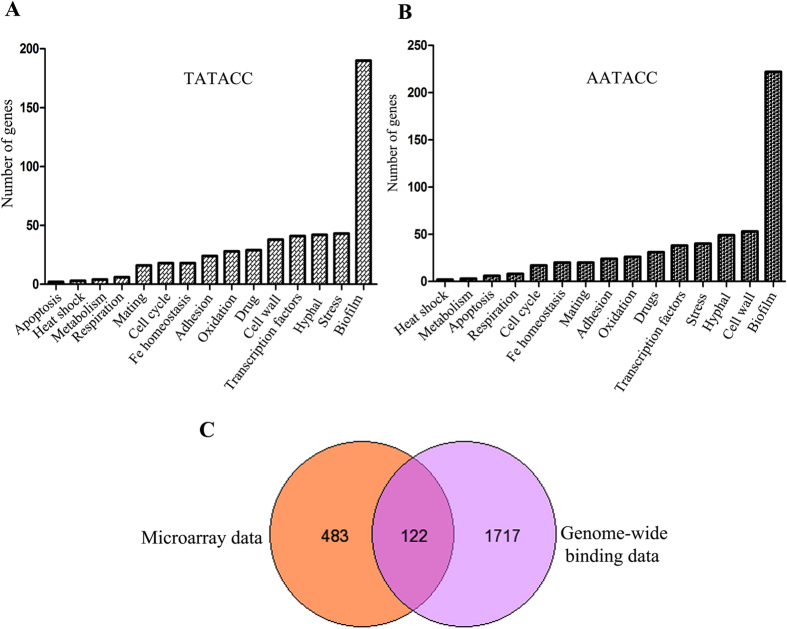
*In silico* genomewide analysis of Zcf32 binding sites shows maximum binding on biofilm gene promoters. (**A,B**) The functional classification of the genes having a Zcf32 binding site (s) in their promoter region is represented. The specific nucleotide sequence mentioned on top of each graph is the Zcf32 binding sequence for which *in silico* genomewide analysis was carried out. The majority of the genes with at least one of the Zcf32 binding sites in the promoter region were found to be biofilm-related. (**C**) A common set of genes which are differentially expressed in YPK102 and possessing the Zcf32 binding site in their promoter region are depicted.

To get an insight into the genome-wide distribution of Zcf32 binding sites, an *in silico* analysis was carried out. Since the mutational analysis data for Zcf32 binding consensus showed that first three positions are not critical for binding of the transcription factor, we considered the 6 bp sequence excluding first three base pairs for genome-wide analysis of Zcf32 binding sites. We searched for four sequences *viz* TATAGC, TATACC, AATAGC and AATACC in the inter-genic regions of the *C. albicans* genome using Patmatch tool available at CGD (www.candidgenome.org)^32^. The presence of these four sequences was further specifically searched in the putative promoter regions. Table 3 shows the summary of the data obtained from the genomewide analysis. *In silico* genome-wide binding analysis of Zcf32 sequences displayed that total 1,970 genes promoters show the presence of Zcf32 recognition sequences (Supplementary sheet 3). The functional categorization of the genes showing the presence of any of the four Zcf32 binding sequences showed that maximum numbers of genes are biofilm development related (p < 0.05)(Fig. 5A,B, Supplementary sheet 4 and Supplementary Fig. S7A,B). Further analysis suggested that a total of 122 genes are common among the differentially expressed genes obtained by the microarray analysis and *in silico* genome-wide binding data for Zcf32 (Fig. 5C). This analysis also implies that out of 607 differentially expressed genes, 122 might be under the direct regulation of *Zcf32* in the planktonic mode of growth at 30 °C.

### ZCF32 directly regulates biofilm-related genes

**Figure 6:**
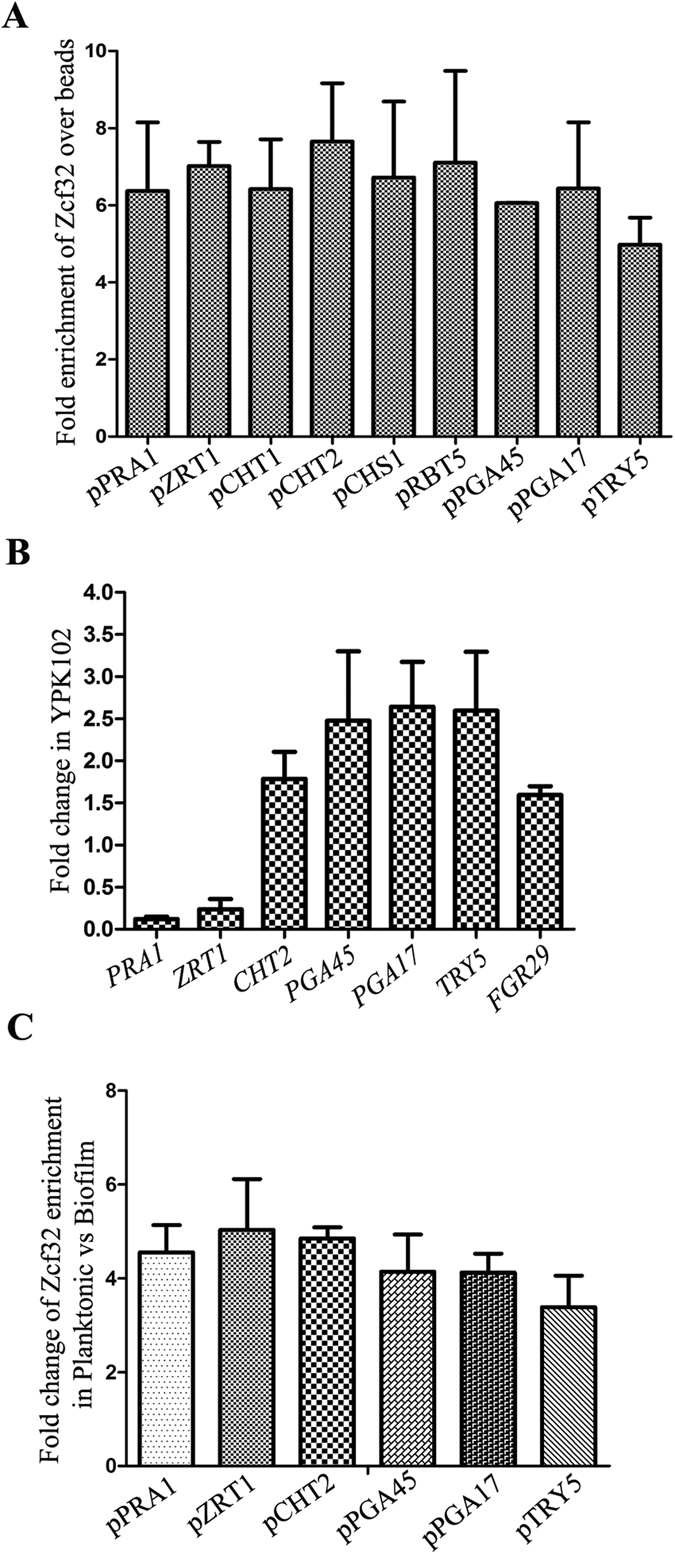
Zcf32 directly regulates the expression of biofilm-induced genes. (**A**) The binding of Zcf32-TAP protein inside the cells on the promoters of biofilm genes was tested by ChIP- qPCR analysis. Zcf32-TAP strain (YPK104) was grown in the planktonic mode of growth at 30 °C and ChIP assay was carried out using antibodies against Protein A. Data are the average of duplicate samples and are representative of two independent experiments. The fold enrichment was calculated over Protein A beads control (mock) by percent input method. Significant enrichment of the transcription factor was observed on the promoters of biofilm-induced genes. (**B**) The expression of biofilm-induced genes was analyzed in SC5314 and YPK102 grown in the biofilm mode of growth. All the genes were normalized to *ACT1* control and then fold change in the expression levels of these genes in YPK102 was calculated over SC5314 control. Results are the average of duplicate samples and are representative of three independent experiments. (**C**) The recruitment of Zcf32-TAP protein on the promoters of biofilm genes was analyzed in the YPK104 cells grown in biofilm as well as in the planktonic mode of growth. Results from two independent experiments are considered. The fold enrichment was calculated over Protein A beads control (mock) by percent input method. Further, the fold change in the binding of Zcf32-TAP on the promoters of biofilm-induced genes was calculated for planktonic versus biofilm condition.

We found out the specific binding sites of Zcf32 by carrying out SELEX. An *in silico* genome-wide analysis of Zcf32 binding sequences showed the presence of these sequences on the promoters of the majority of biofilm genes. We selected a set of genes, *PRA1*, *ZRT1*, *TRY5, CHT1, CHT2, CHS1*, *RBT5, PGA45, PGA17,* and *FGR29* which get differentially expressed in the biofilm mode of growth to study the binding of Zcf32 inside the cells^6^,^30^. To investigate the binding of Zcf32 to the promoter regions of these genes, chromatin immunoprecipitation followed by quantitative PCR (ChIP- qPCR) analysis was carried out with YPK104 (Zcf32–TAP strain). A significant enrichment of Zcf32 was observed on the promoters of the above-mentioned genes which confirm their direct regulation by the transcription factor (Fig. 6A). *ZCF32* positively regulates the expression of *PRA1* and *ZRT1* while negatively regulating the expression of *CHT1, CHT2, CHS1, PGA45, PGA17, TRY5* and *RBT5* as indicated by microarray and ChIP- qPCR data.

The expression of biofilm genes was also analyzed in the SC5314 and YPK102 strains grown in the biofilm mode of growth. It was observed that *PRA1* and *ZRT1* were downregulated in the mutant while *CHT2, PGA45, PGA17,* and *TRY5* showed more expression (Fig. 6B). As we show that *ZCF32* negatively regulates biofilm development, the ChIP- qPCR analysis was carried out from YPK104 cells grown in the biofilm mode of growth. Notably, less recruitment of Zcf32 was observed on the promoters of these genes in the biofilm mode of growth which is represented as fold change in the recruitment of Zcf32 in planktonic versus biofilm mode of growth (Fig. 6C). In order to rule out the possibility that the temperature and the growth medium contribute to the observed increased occupancy of the regulator on the responsive promoters in planktonic mode, the above set of experiments were also carried out at 37 °C and in Spider medium. The results presented in Supplementary Fig. 8A,B essentially show a similar pattern of enrichment of Zcf32. These results establish that *ZCF32* is indeed a negative regulator of biofilm development.

## Discussion

As an opportunistic fungal pathogen, *C. albicans* is capable of producing a highly structured biofilm. Biofilm development is a complex and stepwise process. The biofilm pathway is regulated by a large set of positive regulators and a small set of negative regulators forming a complex network^7^. Our study has added a new regulator in the biofilm gene circuitry. We show that the fungal specific Zn(II)2Cys6 transcription factor, *ZCF32* represses the development of biofilm in *C. albicans*. A comparison of transcriptomes of SC5314 and the *zcf32* mutant strain YPK102 depicts that the transcription factor majorly regulates biofilm genes and the genes involved in hyphae formation. A majority of the genes involved in *ZCF32* mediated regulation of adhesion and filamentation are cell wall proteins. The regulation of these genes by Zcf32 can be direct or indirect as seen with other regulatory factors. By SELEX, we established the specific binding site of Zcf32, suggesting that it functions as a transcription factor. The genome wide *in silico* binding analysis shows that total 1,970 genes exhibit the presence of any of the four Zcf32 binding sequences in their promoter region. Also, the recruitment data for Zcf32 binding inside the cells exhibits that there is more occupancy of Zcf32 on the promoters of biofilm genes in the planktonic mode as compared to the biofilm mode.

The biofilm development in *C. albicans* involves a series of sequential steps. Being the first step, adhesion is essential for the formation of biofilm. Adhesins are instrumental in cell-surface as well as cell-cell adhesion. The cell wall proteins shown to be involved in adhesion are classified into three major families- Als family, Hwp family and Iff/Hyr family^33^. We see the overexpression of adhesins like *CHT2*, *PGA38*, *RBT5* and *SAP4* which belong to Hwp family, in *zcf32* null mutant. It has been reported that the overexpression of *RBT5* in *bcr1/bcr1* mutant can partially rescue the biofilm initiation defect, suggesting its probable role in adhesion^17^. Also, it has been documented that *RBT5* is one of the highly expressed genes in the biofilm mode of growth as compared to planktonic condition^6^,^30^. The transcriptome data also manifested that an adhesion regulator, *TRY5,* a Zn(II)2 Cys6 transcription factor is repressed by *ZCF32*^15^. Thus, our data suggest that *ZCF32* regulates the adhesion step by repressing the expression of the transcription factor, *TRY5* and many cell wall proteins which belong to Hwp family of adhesins.

In addition to adhesins, we show that the major chitinases *CHT1*, *CHT2* and *CHT3* and a chitin synthase *CHS1* are upregulated in YPK102. Chitinases are the cell wall remodelling enzymes. Overexpression of *CHT2* results in hyper-filamentation phenotype^34^. It has also been reported that high biofilm former (HBF) strains have high chitinase activity as compared to low biofilm formers (LBF) which result in autolysis of cells and release of extracellular DNA (eDNA). The eDNA released gets incorporated in the ECM component of biofilm and helps in maintaining its integrity^35^. Thus, we propose that the high chitinase activity as a result of overexpression of chitinases may help in robust biofilm formation by *zcf32* null mutant.

A large set of putative GPI-anchored (PGA) family protein encoding genes, *PGA10, PGA13, PGA17, PGA25, PGA38* and *PGA45* also showed increased expression in YPK102 as compared to SC5314. Except *PGA38* all the mentioned PGA family genes were reported to be induced in either flow and/or spider models of biofilm^6^,^29^,^30^. We found their upregulation in YPK102 suggesting a direct correlation between their higher expression and an enhanced biofilm formation by *zcf32* null mutant. *PGA45* is shown to be involved in Mob2 dependent hyphae formation and in the absence of *ZCF32*, it showed increased expression leading to hyper-filamentation phenotype. *PGA17* is reported to be induced in a mouse model of oropharyngeal candidiasis which can be considered as an *in vivo* biofilm^36^. Increased production of this set of cell wall proteins results in hyper-filamentation and thus increased biofilm formation by the *zcf32* null mutant.

In addition to PGA family proteins, a few other cell wall proteins like *RBT5*, *FGR45*, *FGR29* and *HYR1* were observed to be induced in YPK102. Also, the literature shows that cell wall proteins like *CHT2* and *RBT5* have dual roles in adhesion as well as filamentation. They are shown to be involved in filamentous growth in *C. albicans* and hence the overexpression of these genes in the *zcf32* null mutant possibly facilitates a more robust hyphae formation and hence enhances biofilm development. Thus, *ZCF32* represses filamentation and biofilm formation by repressing the expression of cell wall proteins. Two zinc homeostasis genes *PRA1* and *ZRT1* were found to be repressed in YPK102. They are also reported to be repressed in *zap1/zap1*^9^ and were found to be induced in biofilm condition as compared to planktonic growth^6^. But their exact role in biofilm development is not yet clear.

**Figure 7:**
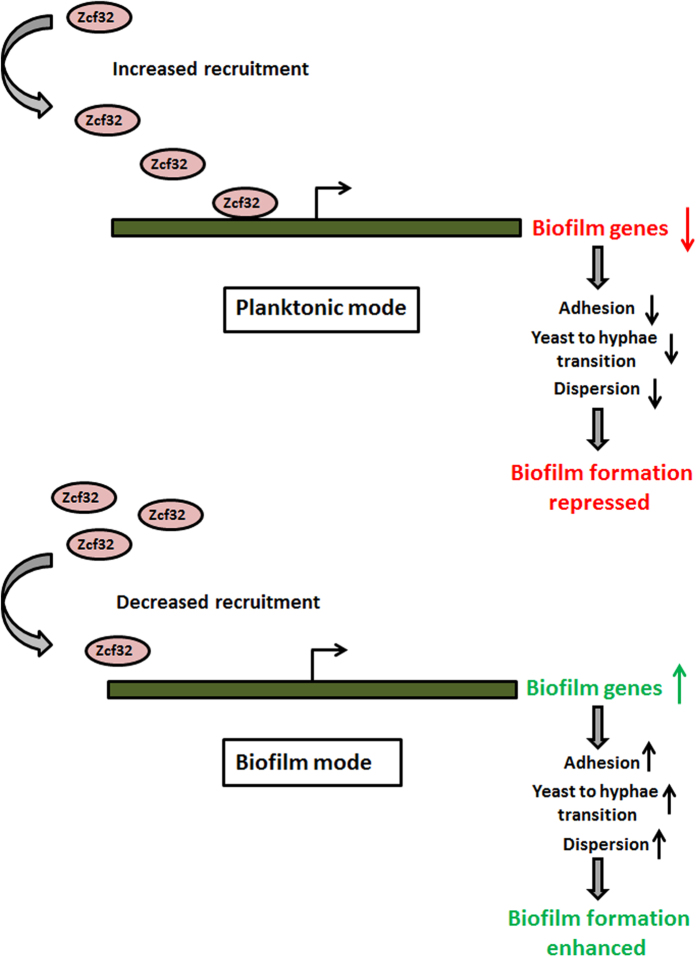
A proposed model for the regulation of biofilm development by *ZCF32.* The transcription factor Zcf32 gets recruited more on the promoters of biofilm-induced genes in the planktonic mode of growth and represses their expression. Less expression of these genes results in less adhesion, yeast to hyphae transition and dispersion which in turn leads to reduced biofilm formation. However, in the biofilm mode of growth, there is decreased binding of Zcf32 on the promoters of biofilm-induced genes resulting in more expression of these genes. The increase in the expression level of biofilm-induced genes advances adhesion and following steps of biofilm development. Thus, we propose that the zinc cluster transcription factor, Zcf32, is a negative regulator of biofilm development in *C. albicans*.

As a consequence of increased recruitment of Zcf32 on promoters of biofilm genes in the planktonic mode of growth the expression of biofilm-induced genes is repressed, resulting in decreased adhesion, yeast to hyphae transition and dispersion. Overall, these changes lead to decreased biofilm formation. Conversely, in the biofilm mode of growth, Zcf32 binding on the promoters of biofilm-induced genes is reduced, resulting in their increased expression. Derepression of genes known to be involved in different stages of biofilm development results in enhanced biofilm formation (Fig. 7). Hence, a gain-of-function phenotype of an enhanced biofilm formation was observed in the case of the *zcf32* null mutant. The genome-wide expression data for planktonic versus biofilm mode of growth do not show any change in the expression level of *ZCF32*^29^,^30^. However, we have observed the differential recruitment of Zcf32 on the promoters of biofilm-induced genes in these two growth conditions implying that there may be different interacting partners of Zcf32 in the two growth conditions. Taken together, our results show the negative regulation of biofilm production in *C. albicans* by a novel transcription factor, *ZCF32.* Its action appears to be mediated by directly controlling adhesion and filamentation processes and indirectly controlling biofilm maturation and dispersion. More importantly, our study adds on a new regulator in the less studied class of negative regulators of biofilm development. As *C. albicans* is primarily a commensal, the negative regulators of biofilm development and other virulence traits might help the organism to maintain the commensal state.

Finally, Zcf32 is a new member of the growing family of transcription factors involved in the regulation of biofilm formation in *C. albicans*. As such, there is an expansion of Zn(II)2Cys6 binuclear cluster transcription factor family in the organism. Total 82 of them are found in *C. albicans* compared to 58 in *Saccharomyces cerevisiae*^23^,^24^,^37^,^38^. Out of these, about 40 of them have been shown to be involved in various stages of biofilm development^6^,^15^,^30^. The role of many of these transcription factors is not yet understood. These transcription factors together with other classes of transcription regulators involved in the biofilm regulation form a formidable regulatory network in determining the switch from the planktonic to biofilm mode of growth. Although the individual roles of a number of transcription factors are elucidated, including the regulation manifested by six master regulators, a complete picture is yet to emerge. Thus, a comprehensive knowledge of the control of the process would require further work using a variety of approaches.

## Methods

### Strains and growth media

The *C. albicans* strains used and generated in this study are listed in Supplementary Table S3. Generation of gene knockout was carried out in SC5314 strain background using SAT1 flipper cassette while for genomic TAP tagging of the gene, SN148 strain was used. Strains were routinely maintained on YPD agar medium (1% yeast extract, 2% peptone, 2% dextrose and 2% agar) or synthetic dropout (SD) agar (0.67% YNB, 2% dextrose and 2% agar) supplied with desired amino acids. Selection of transformants was carried out on YPD + 200 μg/ml nourseothricin while URA3 positive transformants were selected on SD medium lacking uridine. For *in vitro* biofilm assays, strains (pre-inoculum) were first grown in SD + 50 mM galactose medium and then biofilm assays were carried out using Spider medium (1% yeast extract, 1% peptone, 1% mannitol, 0.25% NaCl, 0.1% K_2_HPO_4_).

### Strain construction

The deletion cassette was released from the plasmid pPK928 using KpnI and SacI restriction enzymes. 2–3 μg of the linear cassette was transformed in *C. albicans* SC5314 strain by electroporation^39^ and transformants were selected on YPD containing 200 μg/ml of nourseothricin at 30 °C, yielding the heterozygous deletion strain for *ZCF32*. Further, this heterozygous deletion strain was grown in YP + 2% maltose to excise the SAT1 cassette yielding the strain YPK101. Next, YPK101 was transformed with the same cassette to delete the second copy of *ZCF32.* The *zcf32*/*zcf32* transformants were further screened by PCR and confirmed by Southern blot analysis. This strain was further grown in YP + 2% maltose to yield a homologous deletion strain of *ZCF32* (YPK102) isogenic with SC5314.

#### Total RNA isolation, microarray analysis and quantitative Real Time PCR

Total RNA was extracted from SC5314 and YPK102 cells by Trizol (Sigma-Aldrich) method as per manufacturer’s instructions with slight modification. For growth-dependent expression analysis of *ZCF32,* SC5314 strain was grown as mentioned above. Total RNA was isolated from cells collected at each point of time as mentioned above. RNA samples were quantified using nanodrop machine. 10 μg total RNA of each sample was subjected to DNase I (Roche) treatment (as per manufacturer’s instructions) followed by cDNA synthesis using Superscript III RT (Invitrogen). cDNA was diluted to the desired dilution and the real-time quantitative PCR was carried out using Applied Biosystems machine.

For microarray experiment, SC5314 and YPK102 strains were grown at 30 °C till the start of the stationary phase (24 h) and then harvested. Cell pellets were washed with PBS and then either stored at −80 °C or subjected to total RNA isolation. Total RNA was isolated using Qiagen mini RNA isolation kit. Total RNA from each strain was then subjected to microarray analysis. The detailed protocol of microarray experiment is discussed in the supplementary methods.

#### Biofilm assays.

*In vitro* biofilm assays were carried out as described by others^4^ with slight modification. Strains were grown in synthetic dropout medium provided with 50 mM galactose and amino acids overnight at 30 °C, diluted to an OD_600_ 0.5 in 2 ml of Spider medium and added to a sterile 12-well plate. The 12-well plate was previously treated with bovine serum (Gibco, Invitrogen) overnight at 37 °C and washed with PBS. The plate was incubated at 37 °C for 2 h at 90 rpm for initial adhesion. The wells were washed with 2 ml of PBS twice and 2 ml of fresh Spider medium was added to each well. This plate was incubated at 37 °C for an additional 60 h.

Biofilms formed by the SC5314 and *zcf32* mutant strains were quantified by a modification of a crystal violet assay as described by others^4^,^40^,^41^. Briefly, the biofilm harbouring wells of a 12-well plate were washed twice with 2 ml of PBS and the biofilms were stained with 2 ml of 0.4% aqueous crystal violet solution for 1 h. Afterwards, each well was washed four times with 2 ml of PBS and immediately destained with 2 ml of 95% ethanol overnight. The destained solution was diluted to the desired dilution with 95% ethanol and measured spectrophotometrically at 595 nm. The absorbance values for the controls were subtracted from the values for the test wells to minimize background interference.

The dry mass measurement was carried out as described by Nobile *et al.* with slight modification^9^. After 60 h of growth, biofilms were washed with PBS and then transferred to 0.45 μm filter papers which were pre-weighed. Biofilm material present on filter paper was subjected to filtration using vacuum filter to get rid of the liquid medium, dried in an incubator at 65 °C for 3 h, and weighed. The actual mass of the biofilm was obtained by subtracting the weight of the filter paper from that of the respective dried samples.

Adherence ability of different strains was measured by adherence assay. Strains were grown till initial adhesion step as mentioned above. The wells were washed twice with 2 ml of PBS which was followed by staining of the cells with 0.4% crystal violet for 15 min. Further, each well was washed with PBS four times and immediately destained with 2 ml of 95% ethanol overnight. The destained solution was diluted to the desired dilution with 95% ethanol and measured spectrophotometrically at 595 nm. The absorbance values for the controls were subtracted from the values for the test wells to minimize background interference.

#### SEM and CLSM studies of Biofilm.

SEM analysis was done with a slight modification in the protocol used earlier^42^. Biofilms of wild-type and *zcf32* mutant strains were grown on the human urinary catheter for different periods of times and then processed for SEM imaging. The surface topographies of biofilms were visualized with a scanning electron microscope in high-vacuum mode.

For CLSM analysis, biofilms of different strains were grown on silicon sheets (1 cm X 1 cm) pre-treated with serum. To study the ability of different strains for the yeast to hyphal transition and dispersion, biofilms were grown for 6 h and 48 h respectively, stained with calcofluor white and visualized with a Zeiss LSM 710 Meta confocal laser scanning microscope and images were analyzed using ZEN 2009 software.

#### Selective Evolution of Ligand by Exponential Enrichment (SELEX).

SELEX experiment was carried out with modification in the protocol first described by Feltkamp *et al.*^43^. A pool of 18 mer double-stranded DNA library with 4^18^ different combinations was used for the SELEX experiment. These oligonucleotides were flanked on 5′ and 3′ ends by M13 forward and reverse primers respectively. MBP and Zcf32 ZFN-MBP were purified as discussed in the supplementary method section. 200 ng of the double-stranded DNA library was incubated with 100 μg of MBP and Zcf32 ZFN-MBP for 30 min at 4 °C. Then amylose resin (20 μl bed volume) which was equilibrated with 1X HEPES buffer (20 mM HEPES pH 7.8, 150 mM KCl, 0.1% BSA and 10% glycerol) and pre-treated with salmon sperm DNA was added to the reaction and incubated at 4 °C for 1 h. This was followed by the removal of the supernatant and stepwise washing of beads with 1X HEPES buffer for 5–7 times. The beads were resuspended in 100 μl autoclaved water and boiled for 15 min. The boiled fraction was separated from the beads by centrifugation and 1 μl of this sample was used as a template for the PCR with M13 forward and reverse primers (1–95 °C for 3 min, 2–95 °C for 10 sec, 3–65 °C for 15 sec, 4–72 °C for 15 sec, 5–72 °C for 3 min, steps 2 to 4 were cycled for 20 times). This cycle of different steps was repeated until the saturation was observed in the levels of PCR products. Enriched ds DNA oligonucleotides from the 7^th^ round were cloned in pGEM- T Easy vector (Promega) and clones were confirmed by EcoRI restriction enzyme digestion. Positive clones were sequenced. Eighteen independent sequences were obtained from sequencing of 60 positive clones. The consensus sequence analysis was carried out using MEME analysis tool^44^.

#### EMSA (Electrophoretic Mobility Shift Assay).

Single-stranded 18 bp oligonucleotides O1 and O2 were end labelled using ATP-[γ-^32^P] and T4 polynucleotide kinase (NEB) and then made double stranded by incubating with the complementary oligonucleotides in 1X annealing buffer (10 mM Tris-Cl pH 7.5, 1 mM EDTA and 50 mM NaCl). Labelled double-stranded oligonucleotides were further incubated with Zcf32 ZFN-MBP fusion protein in the presence of 1X HEPES buffer on ice for 30 min. Reaction mixtures were electrophoresed on 6% native PAGE. Gels were exposed to phosphorimaging cassettes for 4 h and scanned using phosphorimager (GE healthcare). For mutational analysis of the Zcf32 binding consensus, EMSA reactions were carried out with 27 variant DNA oligonucleotides (V1 to V27) (Table S1) and DNA oligonucleotide O1 as a control.

#### Chromatin immunoprecipitation (ChIP) assay.

ChIP assays were performed using a protocol described previously^45^ with a few modifications. The ChIP assays were carried out with YPK104 cells grown in YPD medium at 30 °C and in Spider medium at 37 °C both in the planktonic as well as biofilm mode of growth. The cells of YPK104 (ZCF32-TAP tagged strain) grown in different growth conditions were fixed with 1% formaldehyde for 30 min. The reaction was quenched for 15 min at room temperature using glycine to a final concentration of 125 mM. The cell pellet was either used for immunoprecipitation or stored at −80 °C. The detailed protocol is provided in the supplementary methods.

## Additional Information

**Accession number**: The complete data for transcriptome analysis of SC5314 versus YPK102 has been deposited into the NCBI Gene Expression Omnibus (GEO) portal under the accession number GSE76165.

**How to cite this article**: Kakade, P. *et al.* *ZCF32*, a fungus specific Zn(II)2 Cys6 transcription factor, is a repressor of the biofilm development in the human pathogen *Candida albicans. Sci. Rep.* **6**, 31124; doi: 10.1038/srep31124 (2016).

## Supplementary Material

Supplementary Information

Supplementary Dataset 1

Supplementary Dataset 2

Supplementary Dataset 3

Supplementary Dataset 4

## Figures and Tables

**Table 1:** Differentially regulated genes in YPK102 (*zcf32*/*zcf32*).

Gene	Fold change (Log2)	Description
*PRA1*	−2.28	Zinc homeostasis, induced in biofilm
*ZRT1*	−2.49	Zinc homeostasis, induced in biofilm
*CHT1*	2.49	Chitinase
*CHT2*	1.74	Chitinase, Spider biofilm repressed
*RBT5*	2.15	Cell wall protein, involved in haemoglobin utilization, induced in biofilm
*PGA45*	2.47	Cell wall protein, induced in biofilm
*CHS1*	1.9	Chitin synthase, Spider biofilm repressed
*PGA17*	2.35	Cell wall protein, induced in biofilm
*TRY5*	1.45	Adhesin, induced in biofilm
*FGR29*	1.54	Cell wall protein, regulates yeast to hyphal transition

**Table 2:** Sequences of eighteen different oligonucleotides enriched in the SELEX experiment.

Sequence	Frequency of occurrence
TACCCGATATAGCCGATG	58.33%
TACCCGATATAGCGGACG	3.33%
GTACGTTATAGCGGTCAC	1.67%
CGTACGTTATAGCGGTCA	5%
CCGATATAGCCGATGCAT	5%
ACCCGATATAGCCGATGC	1.67%
TCCGGTATGTTCCAGTTG	3.33%
AGAGTTCAAGTAAGTTCC	3.33%
CGTACGTTATAGCGGTCA	3.33%
CCGAATTAGCCGAACGTG	1.67%
CGGTTTCGGCTATTTTGG	1.67%
AGTAAACTTTTCTTTACA	1.67%
CCGGGAAGGATCCCGCTG	1.67%
GAGTTCAAGTAAGTTCCC	1.67%
TGGGAACTTACTTGAACT	1.67%
GTACGTTATAGCGGTCAC	1.67%
TGTTGCCATTTTGTCACC	1.67%
TCCTATAAGTTCCAGTTG	1.67%

The nucleotide sequences of different oligonucleotides with their frequency of occurrence upon sequencing of 60 positive clones are listed.

**Table 3:** Genomewide occurrence of Zcf32 binding sequences obtained by *in silico* analysis.

Sequence	Intergenic region hits	Putative promoter region hits
TATAGC	831	492
TATACC	933	442
AATAGC	971	468
AATACC	1140	568
