# Supplementary material for: ZCF32, a fungus specific Zn(II)2 Cys6 transcription factor, is a repressor of the biofilm development in the human pathogen Candida albicans
Source: Sci Rep. 2016 Aug 8;6:31124. doi: 10.1038/srep31124 (PMC4976313; doi:10.1038/srep31124)
Supplement: Supplementary Information [file srep31124-s1.pdf]

ZCF32, a fungus specific Zn(II)2-Cys6 transcription factor, is a repressor of the biofilm development in the human pathogen _Candida albicans_

Pallavi Kakade [1], Parag Sadhale [1#], Kaustuv Sanyal [2], Valakunja Nagaraja [1, 2*]

[1] Department of Microbiology and Cell Biology, Indian Institute of Science, C.V. Raman Avenue, Bangalore 560012, India

[2] Jawaharlal Nehru Centre for Advanced Scientific Research, Bangalore 560064, India

*For correspondence email at vraj@mcbl.iisc.ernet.in

Ph: + 91 80 23600668, Fax: + 91 80 2360 2697

[#]Deceased on 20 January 2012

METHODS:

DNA manipulation

ZCF32 deletion strain was generated using SAT1 flipper cassette strategy[23]. N- terminus 348 bp and C-terminus 450 bp regions of *ZCF32* ORF were amplified using PK1-PK2 and PK3-PK4 primer pairs **(Supplementary Table S4)** respectively, from SC5314 genomic DNA and cloned in pGEM- T Easy vector (Promega) generating pPK925 and pPK926 vectors. *ZCF32*-NTD (from pPK925) was cloned in pSFS2 vector[23] at the 5' end of the SAT1 flipper cassette (KpnI- XhoI) to generate pPK927. *ZCF32*-CTD (from pPK926) was cloned at NotI- SacI sites in pPK927 which gave rise to pPK928 vector.

For generating the *ZCF32* single re-integrant strain, *ZCF32* ORF with 150 bp upstream region was amplified using PK5-PK6 primer pair from SC5314 genomic DNA and cloned into the pGEM- T Easy vector to generate pPK929. This was sub-cloned in pSFS2 at KpnI- XhoI restriction sites to construct pPK930. *ZCF32* 500 bp downstream region from vector pPK797 (pGEM- T Easy containing 500 bp region downstream of *ZCF32*) was sub-cloned in pPK930 between NotI and SacI sites to generate pPK931. All the plasmids used and generated in this study are listed in **Supplementary Table S2**.

Generation of single re-integrant strain of *ZCF32* from YPK102 (*zcf32/zcf32)*

ZCF32 re-integration cassette was transformed in YPK102 by the method of electroporation. Transformants were selected on YPD containing 200 μg ml^{-1} of nourseothricin at 30°C and were further confirmed by PCR and Southern blot analysis.

Genomic TAP tagging of *ZCF32*

The TAP tag was added to the C-terminus end of *ZCF32* using the method of homologous recombination. TAP tag sequence with URA3 gene was amplified from plasmid pPK335 (Corvey *et al.*, 2005) using the long primers PK9 and PK10 as listed in **Table S4**. The linear PCR product (2-3 μg) was transformed into SN148 strain by the method of electroporation [26]. Selection of the transformants was carried out on SD medium lacking uridine at 30°C. Transformants were further selected by Southern blot analysis.

Expression of TAP-tagged protein was analysed by Western blotting using an antibody against Protein A. All the strains used and generated in this study are listed in **Table S3**.

Growth curve analysis

Growth curve analysis was carried out by optical density (OD) and colony forming units (CFU) methods. SC5314 and YPK102 strains were grown till early log phase (OD_{600} 3-4) and then diluted in YPD broth till OD_{600} 0.1 and grown at 30°C in shaking condition (150 rpm). Cultures were sampled after 3, 6, 8, 10, 12, 14, 24, 48, 60 and 84 h. At each point of time, the cultures were subjected to absorbance measurement (600 nm) and also for plating on YPD agar plates after the desired dilution. Plates were incubated at 30°C till the visible colonies appeared and then CFU was calculated.

Microarray analysis

RNA Quality Control: The concentration and purity of the RNA were evaluated using the Nanodrop Spectrophotometer (Thermo Scientific; 1000). The integrity of the RNAs was analysed on the Bioanalyzer (Agilent; 2100). We considered RNA to be of good quality based on the 260/280 values (Nanodrop), rRNA 28S/18S ratios and RNA integrity number (RIN) (Bioanalyzer)

Labelling and Microarray Hybridization: The samples for Gene expression were labeled using Agilent Quick-Amp labeling Kit (p/n5190-0442). 500 ng each of total RNA were reverse transcribed at 40°C using oligo-dT primer tagged to a T7 polymerase promoter and converted to double stranded cDNA. Synthesized double stranded cDNA were used as a template for cRNA generation. cRNA was generated by in vitro transcription and the dye Cy3 CTP (Agilent) was incorporated during this step. The cDNA synthesis and in vitro transcription steps were carried out at 40°C. Labelled cRNA was cleaned up using Qiagen RNeasy columns (Qiagen, Cat No: 74106) and quality assessed for yields and specific activity using the Nanodrop ND-1000.

Hybridization and Scanning: 600 ng of labelled cRNA sample were fragmented at 60º C and hybridized onto a Genotypic designed C albicans_GXP_8X15k (AMADID No: 26377) arrays. Fragmentation of labelled cRNA and hybridization were done using the Gene Expression Hybridization kit (Agilent Technologies, In situ Hybridization kit, Part Number 5190-0404). Hybridization was carried out in Agilent's Surehyb Chamber at 65º C for 16 h. The hybridized slides were washed using Agilent Gene Expression wash buffers (Agilent Technologies, Part Number 5188-5327) and scanned using the Agilent Microarray Scanner (Agilent Technologies, Part Number G2600D).

Feature Extraction: Data extraction from Images was done using Feature Extraction software Version 11.5 of Agilent.

Microarray Data Analysis: Images were quantified using Feature Extraction Software (Version-11.5 Agilent). Feature extracted raw data was analysed using GeneSpring GX software from Agilent. Normalization of the data was done in GeneSpring GX using the 75th percentile shift. Percentile shift normalization is a global normalization, where the locations of all the spot intensities in an array are adjusted. [This normalization takes each column in an experiment independently, and computes the nth percentile of the expression values for this array, across all spots (where n has a range from 0-100 and n=75 is the median). It subtracts this value from the expression value of each entity and fold expression values were obtained with respect to Specific control Samples]. Significant genes up and down regulated showing 0.6 fold (log 2) and above within the samples with respect to control sample were identified. Differentially regulated genes were clustered using hierarchical clustering based on Pearson coefficient correlation algorithm to identify significant gene expression patterns.

Protein Expression and Purification

MBP and Zcf32-ZFN-MBP recombinant proteins were expressed in *E. coli* BL21 pLysS cells and the cell lysates were prepared by sonication using lysis buffer (10 mM Tris-Cl pH 7.5 and 150 mM NaCl). The supernatant was separated by centrifugation and allowed to bind with 100 µl of 50% slurry of amylose beads (NEB) for 3 h at 4°C. This was followed by washing of beads with 20 ml of lysis buffer. MBP and

Zcf32 ZFN-MBP proteins were eluted from the column using a buffer (10 mM Tris-Cl pH 7.5, 150 mM NaCl, 10 mM maltose). All the elutes were pooled and subjected to dialysis in buffer (10 mM Tris-Cl pH 7.5, 150 mM NaCl and 50% glycerol) at 4ºC. The dialyzed proteins were checked for purity by running on 8% SDS-PAGE. Protein concentrations were estimated by Bradford's method.

Chromatin immunoprecipitation assay

Formaldehyde cross-linked cells were resuspended in resuspension buffer (0.2 mM Tris-HCl pH, 9.4 and 10 mM DTT) and incubated at 30°C for 15 min on a shaker at 150 rpm. Cells were once washed with spheroplasting buffer (1.2 M Sorbitol and 20 mM Na-HEPES, pH 7.5). Further, spheroplasting (95%) was performed using lyticase (Sigma) at 30°C and 80 rpm. After getting 95% spheroplast formation, spheroplasting was stopped by adding ice-cold post spheroplasting buffer (1.2 M Sorbitol, 1 mM $MgCl_2$ and 20 mM Na-PIPES pH 6.8). Spheroplasts were subsequently washed with ice-cold 1× PBS, Buffer I (0.25% TritonX-100, 10 mM EDTA, 0.5 mM EGTA and 10 mM Na-HEPES pH 6.5), Buffer II (200 mM NaCl, 1 mM EDTA, 0.5 mM EGTA, 10 mM Na-HEPES pH 6.5) and finally resuspended in extraction buffer (140 mM NaCl, 1 mM EDTA, 50 mM K-HEPES, 0.1% sodium deoxycholate and 1% Triton X-100, pH 7.5) with protease inhibitor cocktail (Roche). Next, sonication was performed to get sheared chromatin fragments of an average size of 300–700 bp by SONICS Vibra cell sonicator. The soluble fraction of sheared chromatin was obtained by centrifuging the sonicated solution at 12,000 rpm for 30 min at 4°C. About 1/10th of total soluble chromatin (300 µl) was processed separately as total input DNA. It was subjected to reverse crosslinking followed by RNaseA (Sigma) and ProteinaseK (Sigma) treatment. The DNA was extracted with an equal volume of phenol / chloroform /isoamyl alcohol (25:24:1) in the presence of 0.4 M LiCl and precipitated with ethanol.

Rest of the soluble chromatin solution was diluted 5.7-fold with IP dilution buffer (167 mM NaCl, 1.1 mM EDTA, 1.1% Triton X-100 and 167 mM Tris–HCl, pH 8.0) and divided equally into two tubes. Rabbit anti-Protein A antibody was added to a final concentration of 8 µg ml^{-1} in one tube (+Ab) and no antibody (−Ab) was added to the other. The tubes were slowly rotated overnight at 4°C. A slurry of Protein A-sepharose beads (Sigma) which were blocked with BSA (500 µg ml^{-1}) and ss DNA (100 µg) overnight at 4°C was added to + Ab and – Ab tubes and the tubes were again rotated overnight at 4°C.

Next, beads were sequentially washed twice in 12.5 ml of extraction buffer, and once each in 12 ml of extraction buffer containing 500 mM NaCl and LiCl wash buffer (10 mM Tris-HCl, pH 8, 250 mM LiCl, 0.5% sodium deoxycholate and1 mM EDTA) and TE. Beads were subjected to elution of IP complexes in elution buffer (1/10 volume of IP dilution buffer) at 65°C overnight. This was followed by treatment with RNaseA and Proteinase K. The DNA was extracted with an equal volume of phenol/chloroform/isoamyl alcohol (25:24:1) in the presence of 0.4 M LiCl and precipitated with ethanol.

Oligonucleotide	Sequence
O1	TACCCGATATAGCCGATG
V1	TACCAGATATAGCCGATG
V2	TACCCTATATAGCCGATG
V3	TACCCGCTATAGCCGATG
V4	TACCCGAGATAGCCGATG
V5	TACCCGATCTAGCCGATG
V6	TACCCGATAGAGCCGATG
V7	TACCCGATATCGCCGATG
V8	TACCCGATATATCCGATG
V9	TACCCGATATAGACGATG
V10	TACCGGATATAGCCGATG
V11	TACCCCATATAGCCGATG
V12	TACCCGTTATAGCCGATG
V13	TACCCGAAATAGCCGATG
V14	TACCCGATTTAGCCGATG
V15	TACCCGATAAAGCCGATG
V16	TACCCGATATTGCCGATG
V17	TACCCGATATACCCGATG
V18	TACCCGATATAGGCGATG
V19	TACCTGATATAGCCGATG
V20	TACCCAATATAGCCGATG
V21	TACCCGGTATAGCCGATG
V22	TACCCGACATAGCCGATG
V23	TACCCGATGTAGCCGATG
V24	TACCCGATACAGCCGATG

V25	TACCCGATATGGCCGATG
V26	TACCCGATATAACCGATG
V27	TACCCGATATAGTCGATG

Table S1. Sequences of oligonucleotide 1 and different variant oligonucleotides (V1 to V27) used for mutational analysis of Zcf32 binding consensus are listed. Specific mutated base in each sequence is highlighted in red colour.

**Plasmid**	**Original vector**	**Description**	**Reference**
pSFS2	-	SAT1 flipper cassette containing vector for gene deletion in *C. albicans*	(Reuss *et al.*, 2004)
pPK925	pGEM-teasy	*ZCF32* NTD (348bp) cloned in pGEM T Easy	This study
pPK926	pGEM-teasy	*ZCF32* CTD (450bp) cloned in pGEM T Easy	This study
pPK927	pSFS2	*ZCF32* NTD cloned in pSFS2 at KpnI/XhoI sites	This study
pPK928	pPK927	*ZCF32* CTD cloned in pPK927 at NotI/SacI sites	This study
pPK929	pGEM-teasy	*ZCF32* ORF with 150bp upstream region cloned in pGEM teasy	This study
pPK930	pSFS2	*ZCF32* ORF with 150bp upstream region subcloned in pSFS2	This study
pPK931	pPK930	*ZCF32* 500bp downstream region cloned in pPK930 at NotI/SacI sites	This study
pPK335	-	A vector containing TAP tagging cassette along with a URA3 selection marker	(Corvey *et al.*, 2005)

Table S2. List of plasmids used and generated in the study

Strain	Genotype	Comment	Reference
SC5314	Clinical isolate	Wild type	(Gillum *et al.*, 1984)
zcf32	*ZCF32/zcf32*::FRT	*ZCF32* heterozygous deletion with flipped SAT1 cassette	This study
zcf32/*zcf32*	*zcf32*::FRT/*zcf32*::FRT	*ZCF32* homozygous deletion strain with flipped SAT1 cassette	This study
zcf32/*zcf32* ::*ZCF32*	*zcf32*::FRT/ *ZCF32*::FRT	*ZCF32* reintegrated at its native locus with flipped SAT1 cassette	This study
SN148	*arg4Δ/arg4Δ leu2Δ/leu2Δ his1Δ/his1Δ ura3Δ*::imm434/*ura3Δ*:: imm434 iro1Δ::imm434 /iro1Δ::imm434	Provides selection for Arg, Leu, His and Ura. Derived From SN76	(Noble & Johnson, 2005)
ZCF32-TAP	*ZCF32*::*URA3*$^{+}$	*ZCF32* TAP tagged at its C -terminus	This study

Table S3: List of strains used and generated in the study

**Primer**	**Details**	**Sequence**
PK1	*ZCF32* NTD F	5'CCCCTCGAGAATGGAGGAAAAGAAGAAAATCC 3’
PK2	*ZCF32* NTD R	5' GGGCTGCAGTGGAGATTTTATTGACTCT 3'
PK3	*ZCF32* CTD F	5’ GACTTGCCACAAGAAACAGTT 3’
PK4	*ZCF32* CTD R	5’ TTACAACAATGTTAGATCAACACC 3’
PK5	*ZCF32* 150bp UP F	5’ CGAGGTACCTTCATATCTCTTCCCTTCCCTT 3’
PK6	*ZCF32* ORF R	5’ CAGCAATACCTGGGAACATGG 3’
PK7	*ZCF32* 500bp DOWN F	5’CCCGAGCTCTTTCTCAAATATTCAATAGTTTGTT 3’
PK8	*ZCF32* 500bp DOWN R	5’ TATGCGGCCGCTCGAGAGCAACGCGTAAATAT 3’
PK9	*ZCF32* TAP	5’CAATGCCCGAAAGGGTTTGCAAATCATGAAA

	tag F	CGGTGTTGGAAAACGAACGAAGATTGGATAACT GCGGCTGAAAAAATTGGTGTTGATCTAACATTGT TGGGAGGATCCATGGAAAAGAGAAGA 3’
PK10	*ZCF32* TAP tag R	5’TCTTGATTTCAGAATTTTTAAAGCTCTT TCTGTTTGTTCTGTATTAAGGTAAAATGCA TGACTAAAAAAAAACATTTTATATTTACGC GGTTGATCTCGATCGATGAATTCGAGCTCGTTC 3’
PK11	*ZCF32* 500bp UP F	5’CGCTAGCACCTGTAATGTAC 3’
PK12	pMal2 R	5’ CCCTCGAGCATTGTAGTTGATTATTAGTTAAACC 3’
PK13	NAT Mid R	5’ CATTTTTATACCGTGTCTTCGTCTATC 3’
PK14	*ZCF32* int F	5’ GCAATTTGATAGGTGATACCTT 3’
PK15	*ZCF32* int R	5’ CAATTGGTCAATCACGATATCG 3’
PK16	M13 F	5' GTAAAACGACGGCCAGT 3'
PK17	M13 R	5' GGAAACAGCTATGACCATG 3'
PK18	*ACT*1 F	5’CGTTGTTCCAATTTACGCTGG 3’
PK19	*ACT1* R	5’ CAGCAATACCTGGGAACATGG 3’
PK20	*ACT1* qRT F	5’CTCCAGAAGCTTTGTTCAGAC3’
PK21	*ACT1* qRT R	5’ CAGCAATACCTGGGAACATGG 3’
PK22	*ZCF32* qRT F	5’CAGTTTTTTGAACAAAGGTTCC 3’
PK23	*ZCF32* qRT R	5’ CAGCAATACCTGGGAACATGG 3’
PK24	*CHT1* qRT F	5’ CCTGTTGCTGCTACTACTAC 3’
PK25	*CHT1* qRT R	5’ TTGTAGCATTTGGCTGCCCA 3’
PK26	*CHT2* qRT F	5’ GCACCAAATACGTCACCATTG 3’
PK27	*CHT2* qRT R	5’ GAAGGCAAAGGCAGCCAATAA 3’
PK28	*CHT3* qRT F	5’ CCCAACTGATACTGAAACTAC 3’
PK29	*CHT3* qRT R	5’ GTTGTAAGGGTGCGAAGAGT 3’

PK30	*CHS1* qRT F	5'CTGACAAGAGCCAACACTGC 3'
PK31	*CHS1* qRT R	5' CGCCTCTTGATGGTGATGAT 3'
PK32	*RBT5* qRT F	5' CTGCTAAAGAAACCACTGCTG 3'
PK33	*RBT5* qRT R	5'GCTTCAACGGAAACAGAAGC 3'
PK34	*PRA1* qRT F	5' GCTTTGGATGTGTATGCATATG 3'
PK35	*PRA1* qRT R	5' CTAGGGTTGCTATCGGTATGTG 3'
PK36	*ZRT1* qRT F	5' GGTAATGACCCCTCTACTTTAA 3'
PK37	*ZRT1* qRT R	5'GAGCAACATTCCCAAAATCAAAC 3'
PK38	*PGA45* qRT F	5' CATCTGCTACTGCTACTGCT 3'
PK39	*PGA45* qRT R	5' GTTTATGTAATGTGGGTGTGTG 3'
PK40	*PGA17* qRT F	5' GTACCATTACATCCTTGCCAG 3'
PK41	*PGA17* qRT R	5' ACCATAAGAGGAACCGGCAT 3'
PK42	*FGR29* qRT F	5' CCTCATCTCCACATTCTAGCA 3'
PK43	*FGR29* qRT R	5' GCTGGATTCGAACTCATCTTC 3'
PK44	*TRY5* qRT F	5' CCATAACCACAATAGTCGCAA 3'
PK45	*TRY5* qRT R	5' CTGTGCCAAGATCCCTAATG 3'
PK46	p*PRA1* ChIP F	5'CAGTATCACGGGCATTGACT 3'
PK47	p*PRA1* ChIP R	5'GACAATAACCATGACGCTGT 3'
PK48	p*ZRT1* ChIP F	5' CTTGCATCTGGTATCTCTTGG 3'
PK49	p*ZRT1* ChIP R	5' CTCCGTGGTTGCTAATCTGTA 3'
PK50	p*CHT2* ChIP F	5' CTAACCCCAAGTCATTCGTTA 3'
PK51	p*CHT2* ChIP R	5' AACGTTCAATGGATCTGGAAAC 3'
PK52	p*RBT5* ChIP F	5' TCCACAGAAGACCCCCATTA 3'
PK53	p*RBT5* ChIP R	5'CGTAGCCCCTAACACCAAAA 3'
PK54	p*PGA45* ChIP F	5' GTTAAACGATACCCAACAATTTC 3'
PK55	p*PGA45* ChIP R	5' GTTTATGTAATGTGGGTGTGTG 3'
PK56	p*PGA17* ChIP F	5' TTTGGAGTATCATCTTGTTTTCC 3'

PK57	p*PGA17* ChIP R	5’ CCTTTGGTAGTCTTTGATGCTT 3’
PK58	p*TRY5* ChIP F	5’ GCCACCAATACAAGAAGACTTT 3’
PK59	p*TRY5* ChIP R	5’ GTTTAAGGTTGTTGATTGCGTG 3’

Table S4. List of primers used in the study

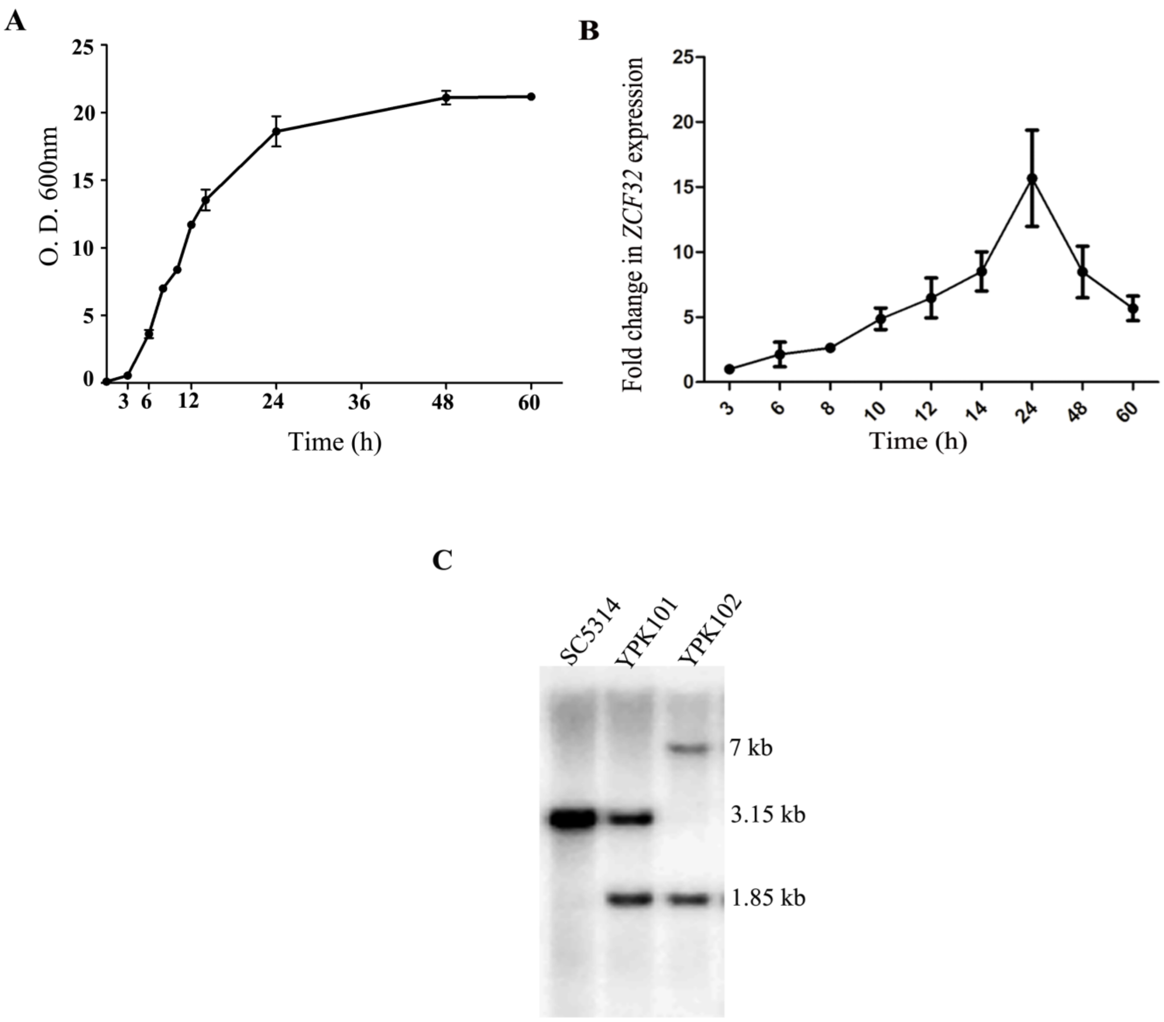

Figure S1. A) Growth curve analysis of SC5314 strain of *Candida albicans* over the period of 60 h in YPD at 30°C. **B)** Expression analysis of *ZCF32* in the growth phase-dependent manner in SC5314 grown over the period of 60 h in YPD at 30°C. **C)** Confirmation of *ZCF32* complete knockout strain by Southern blot analysis. Expected pattern with PvuII digestion of the genomic DNA: SC5314 - 3.15 kb, YPK101- 3.15 kb and 1.85 kb, YPK102 (without flipped cassette) – 7 kb and 1.85 kb.

A

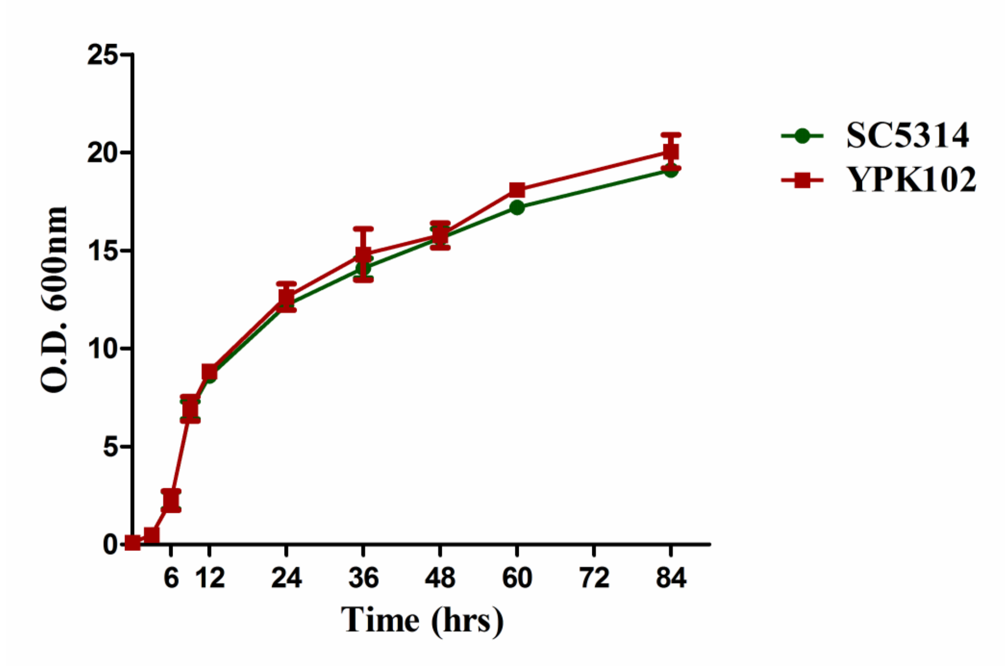

B

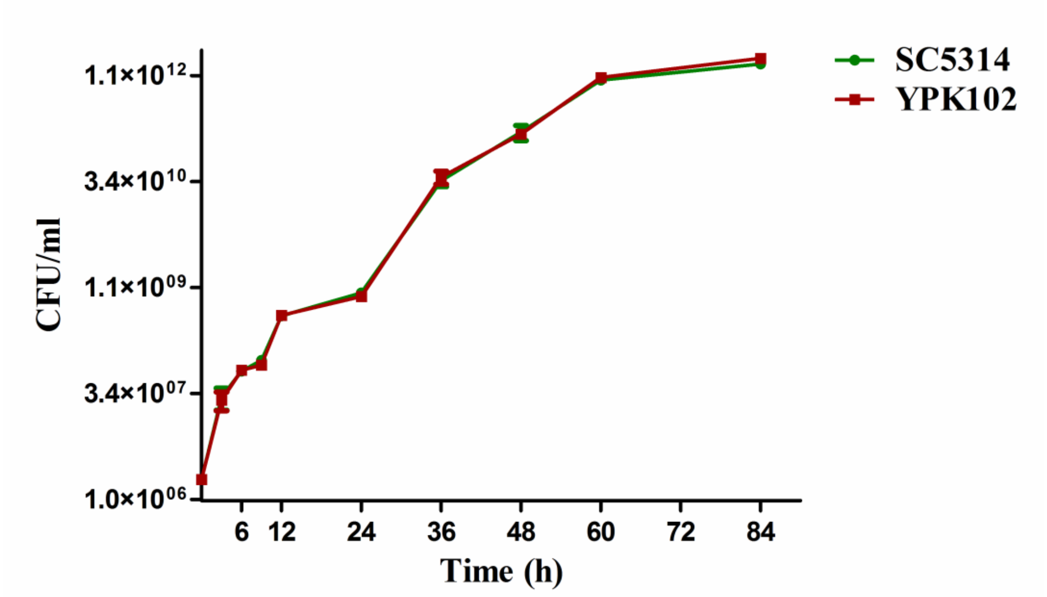

Figure S2. A) Growth curve analysis of SC5314 and YPK102 (*zcf32/zcf32)* strains by optical density method over the period of 84 h. Cell density was measured as absorbance at 600 nm. **B)** Growth patterns of SC5314 and YPK102 strains were compared by CFU analysis done over the period of 84 h. Cells from each indicated time points were plated on YPD agar plates and then incubated at 30°C for 2 days and CFU was calculated as CFU/ml.

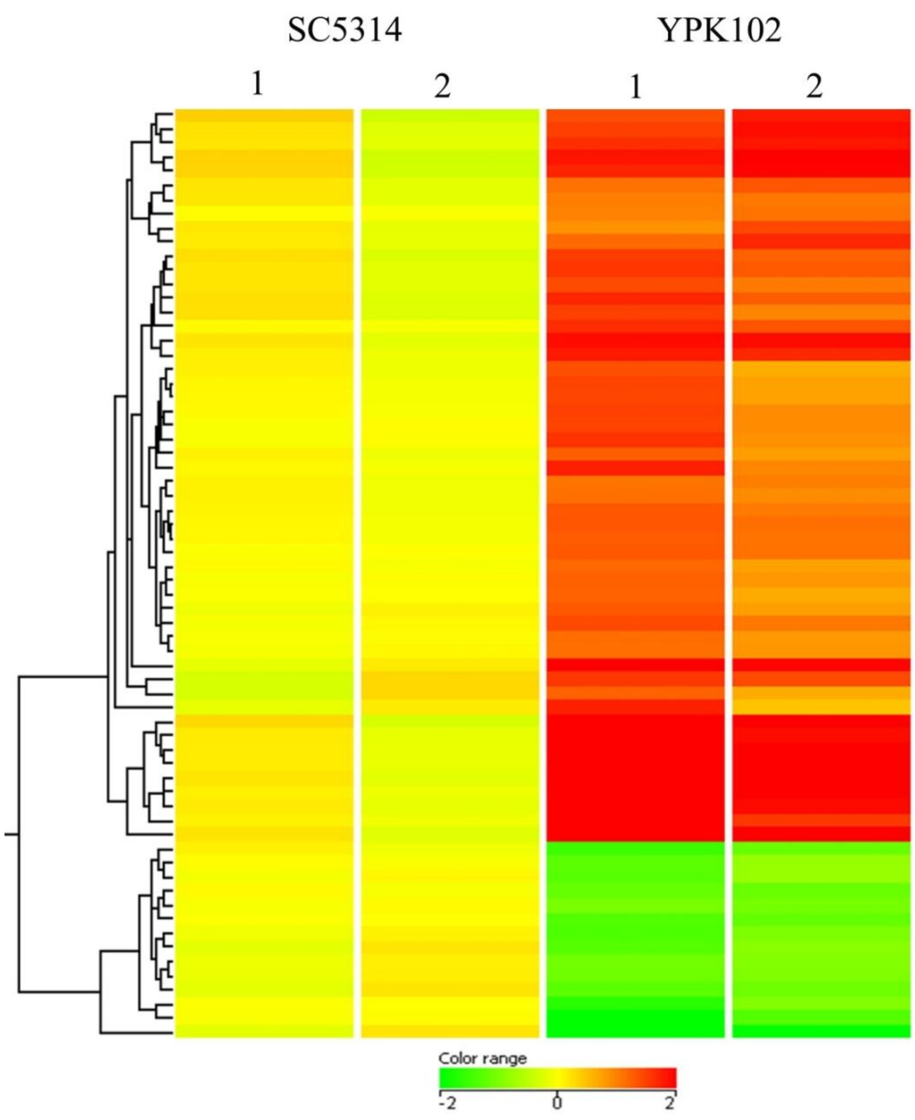

Figure S3: Transcriptomes of SC5314 (wild-type) and YPK102 (*zcf32/zcf32)* were compared by microarray analysis. Both the strains were grown in YPD at 30°C till the start of the stationary phase for the microarray experiment. Expression data from wild-type and mutant is illustrated as the heat map. Gene expression values are represented in the form of colour coded scale as shown at the bottom of the heat map. Total 607 genes were found to be differentially regulated in the mutant compared to the wild-type. Out of which 428 genes were significantly upregulated and 169 were downregulated (Fold change ≥ 1.5, p-value ≤ 0.05).

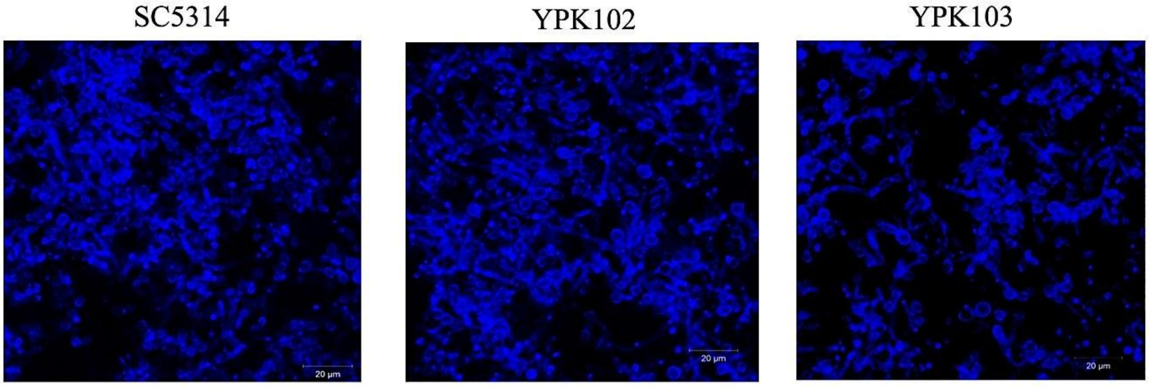

Figure S4. 6 h old biofilms formed by wild-type and mutant strains on silicon sheets were stained with calcofluor white and scored for the presence of hyphal cells by CLSM analysis. YPK102 showed the presence of more hyphal cells compared to SC5314 and YPK103 strains suggesting its negative role in yeast to hyphae transition.

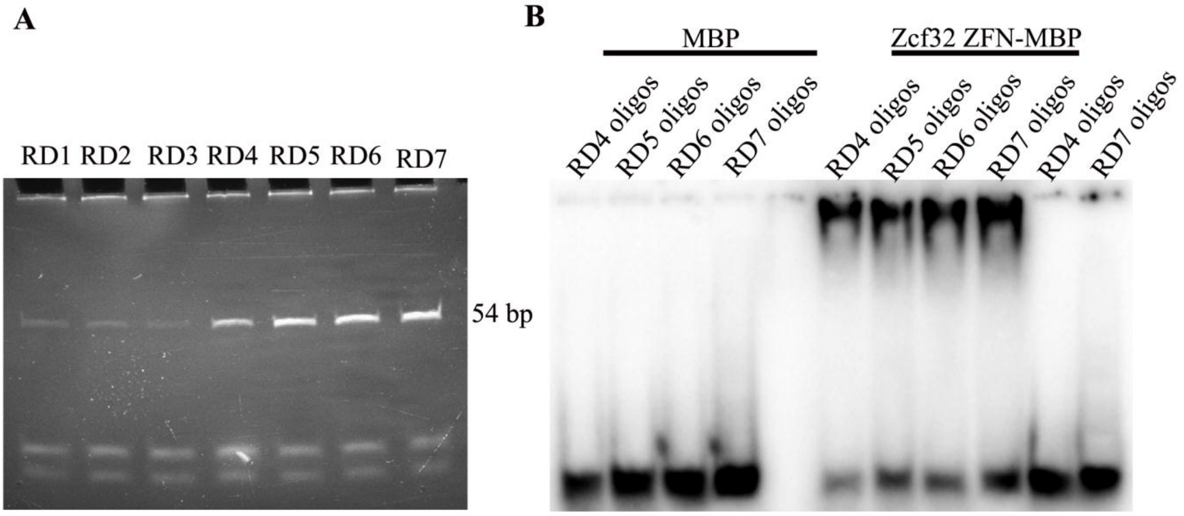

Figure S5. A) A native PAGE gel showing the enrichment of double-stranded DNA from first to seventh rounds of SELEX. **B)** Specific binding of Zcf32 ZFN-MBP to the SELEX enriched oligonucleotides was confirmed by EMSA. Fourth to seventh round SELEX enriched 54 bp double-stranded DNA oligonucleotides showed the specific binding to Zcf32 ZFN-MBP while no shift was observed with only MBP.

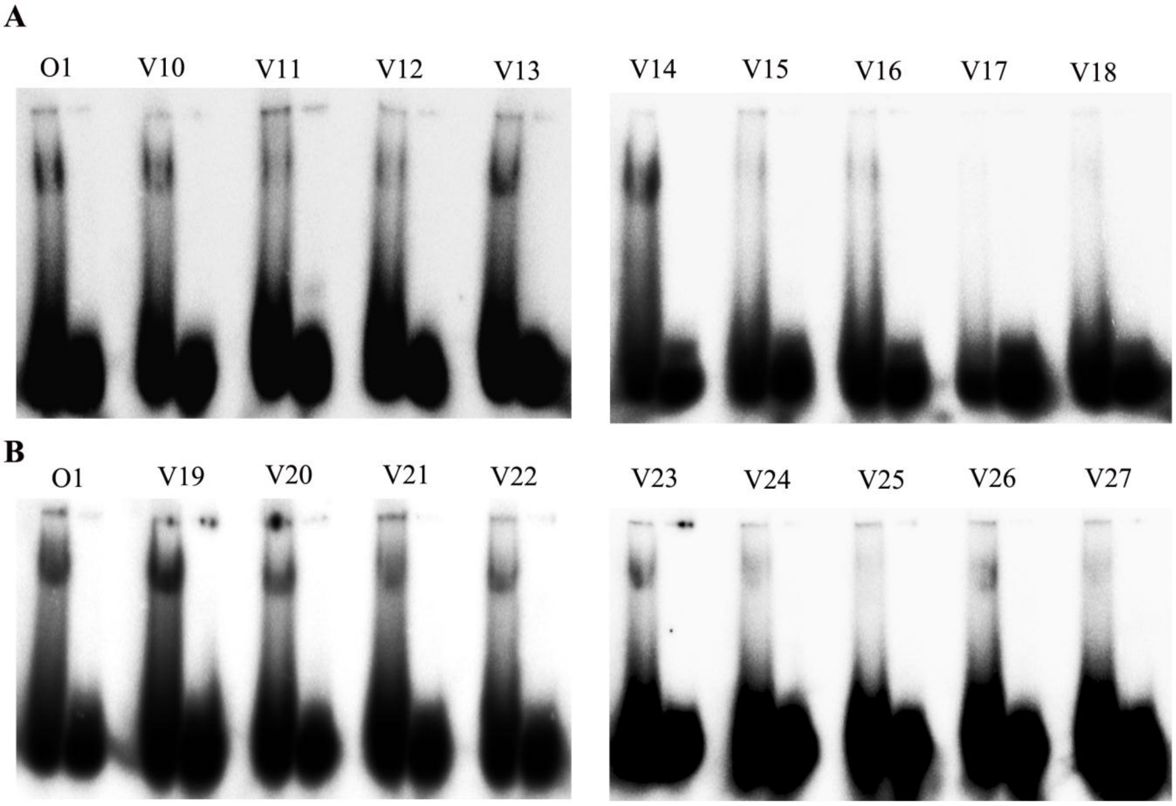

Figure S6. A) EMSA gels for the binding of Zcf32 ZFN-MBP protein with O1 and variant oligonucleotides from V10 to V18. **B)** EMSA gels showing the specific binding of the fusion protein with O1 and variant oligonucleotides from V19 to V27.

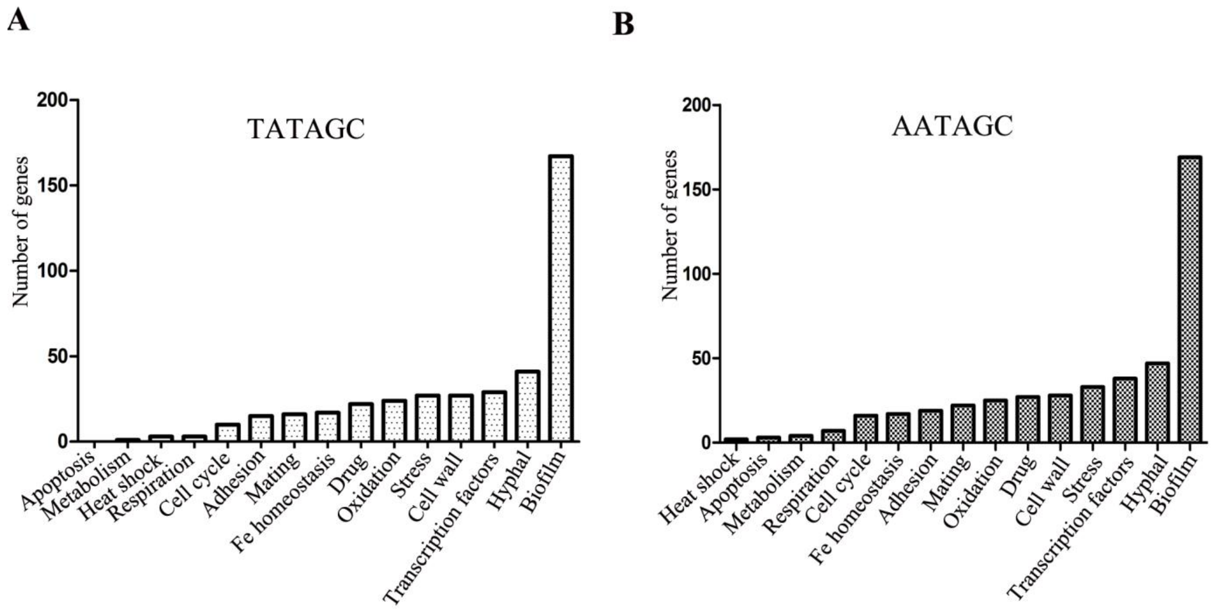

Figure S7. A and B) The functional classification of the genes having Zcf32 binding site/s (TATAGC and AATAGC) in their promoter region is represented. The majority of the genes with either of the Zcf32 binding sites in the promoter region were found to be biofilm-related but the p-value obtained was $p>0.05$.

A

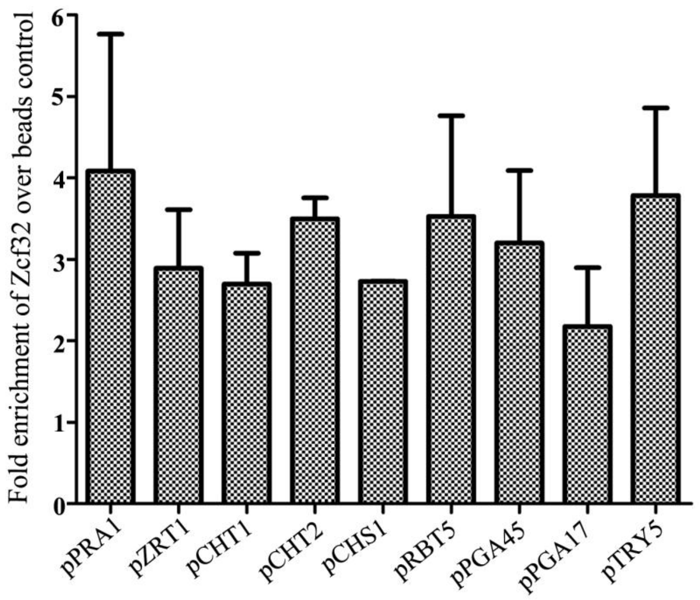

B

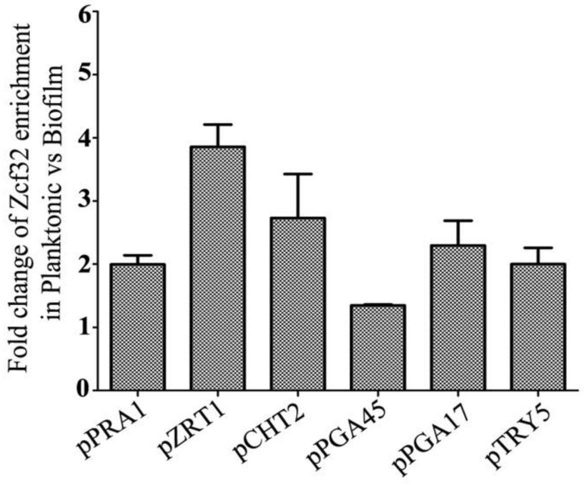

Figure S8: Zcf32 occupancy on the promoters of biofilm-induced genes

A) The occupancy of Zcf32 was studied in the YPK104 cells grown in Spider medium at 37°C in the planktonic mode of growth. Data are the average of duplicate samples and are representative of two independent experiments. The fold enrichment was calculated over Protein A beads control (mock) by percent input method. Significant enrichment of Zcf32 was observed on the responsive promoters.

B) Zcf32 recruitment on the promoters of biofilm-induced genes was compared in the YPK104 cells grown in Spider medium at 37°C both in the planktonic as well as the biofilm mode of growth. Data are

the representative of two independent experiments. Zcf32 displayed more recruitment on the responsive gene promoters in the planktonic mode compared to the biofilm mode of growth.
